# A Dual-Branch Frequency-Aware Attention Framework for Rare Neurological Disease Classification from Brain MRI

**DOI:** 10.3390/diagnostics16111749

**Published:** 2026-06-05

**Authors:** Madallah Alruwaili, Mahmood A. Mahmood

**Affiliations:** 1 Department of Computer Engineering and Networks, College of Computer and Information Sciences, Jouf University, Sakaka 72441, Aljouf, Saudi Arabia; 2 Department of Information Systems, College of Computer and Information Sciences, Jouf University, Sakaka 72441, Aljouf, Saudi Arabia; mamahmood@ju.edu.sa

**Keywords:** rare neurological diseases, brain MRI, RareNeuroXNet, multi-branch deep learning, frequency-domain learning, FFT, CBAM attention, DenseNet121, cross-validation, calibration, Grad-CAM, medical image classification

## Abstract

**Background:** Rare neurological diseases are challenging to diagnose from brain MRI because of their low prevalence, heterogeneous imaging patterns, and limited annotated datasets. Deep learning may support image-level recognition, but results from curated datasets without complete patient-level identifiers require cautious interpretation. **Objectives:** This study proposes RareNeuroXNet, a frequency-aware multi-branch attention framework for image-level classification of rare neurological diseases from brain MRI. The objective was to assess whether combining global anatomical, local fine-grained, and frequency-domain representations improves benchmark performance, calibration, and interpretability. **Methods:** RareNeuroXNet uses three complementary branches: a global branch for whole-image representation, a local branch for regional feature extraction, and an FFT magnitude-based frequency branch. Features are refined using CBAM attention, fused, and classified through a fully connected head. The model was evaluated on a balanced curated dataset with five rare neurological disease classes using five-fold cross-validation, ablation analysis, calibration metrics, internal baseline comparison, paired testing against DenseNet121 local-only, and Grad-CAM visualization. MCND was also used as a complementary cross-dataset neurological MRI benchmark, not as same-task external validation. **Results:** RareNeuroXNet achieved strong image-level internal benchmark performance, with accuracy of 0.9924±0.0061, macro F1-score of 0.9924±0.0061, macro AUROC of 0.9998±0.0002, and macro AUPR of 0.9992±0.0007. Calibration was favorable, with ECE of 0.0052±0.0029 and NLL of 0.0276±0.0159. Ablation results showed that the local branch was the dominant contributor, while FFT and CBAM provided supportive refinement. Compared with DenseNet121 local-only, RareNeuroXNet showed modest classification gains and clearer calibration improvements. **Conclusions:** RareNeuroXNet demonstrated strong controlled image-level benchmark performance with high discrimination, stable cross-validation behavior, favorable calibration, and Grad-CAM interpretability. However, possible correlated slices, duplicate images, or subject overlap cannot be excluded. Future work should use patient-level, same-task, multi-center external validation and 3D multimodal MRI analysis.

## 1. Introduction

Rare diseases represent a major global health challenge, although each individual disease affects only a small proportion of the population. Orphanet estimates that rare diseases, taken as a group, affect hundreds of millions of people across the globe, and many rare diseases remain difficult to diagnose because of heterogeneous phenotypes, limited expert availability, and the scarcity of complete clinical and imaging data. These difficulties are exacerbated in the neurological setting, where radiological appearances may overlap, and there is great clinical variation and expert interpretation of brain imaging [1].

Magnetic resonance imaging (MRI) is one of the most important noninvasive tools for neurological assessment because it provides high soft-tissue contrast and detailed structural information. Over the past few years, deep learning has emerged as a key tool for the analysis of neurological diseases using MRI images, particularly in the field of classification of brain tumors, staging of Alzheimer’s disease, and other widely used MRI applications in neurological disorders. CNNs, transformer-based models, and hybrid architecture have demonstrated strong capacity for learning discriminative imaging features from MRI. These models, however, are not widely applicable due to the small size of medical imaging datasets, the variability of scanners and acquisition protocols across them, and the susceptibility of medical imaging data to domain shifts. In addition, model interpretability, probability calibration, and validation under independent patient-level conditions are important requirements for trustworthy medical AI systems [2].

MRI-based classification research has progressed rapidly since 2020. Pre-trained convolutional neural network models like ResNet, DenseNet, EfficientNet, and VGG were used for early transfer learning with limited labeled datasets, and these models have shown effective transferability to medical imaging tasks. Subsequent research included attention mechanisms, multimodal fusion, image enhancement, transformer modules, and ensemble methods for enhancing the representation of subtle lesions, long-range spatial dependencies, and inter-class variation. Despite these improvements, most deep learning studies on MRI images are still primarily devoted to typical problems in clinical neurological imaging such as brain tumor classification or Alzheimer’s disease staging, while the classification of rare neurological diseases is still relatively under-explored [3].

A major methodological challenge is that high image-level performance does not necessarily imply clinical generalization in MRI-based deep learning. Medical imaging datasets can have several slices, scans or similar visual representations from the same examination or patient. Performance of the model can be artificially boosted if correlated samples are split and distributed among the training, validation, and testing partitions. Brain MRI classification studies have shown that slice-level splitting can introduce data leakage and produce overly optimistic results, whereas patient-level splitting provides a more reliable evaluation [4,5]. This is particularly relevant in rare disease data sets, where the number of subjects is often small, and public data sets might not contain all the patient identifiers.

This issue is particularly relevant because rare neurological diseases often exhibit subtle, localized, and heterogeneous MRI patterns. These factors pose challenges for model development due to the scarcity of annotated datasets and the rarity of diseases that require methods to learn informative representations from relatively small and focused datasets. A scoping review of deep learning in rare disease research found that there is literature on rare neurological diseases, but it is less developed than the literature on applications in rare neoplastic and genetic diseases, and that CNN-based image analysis is the most frequent approach used in these applications [6]. Surveys of neurological diagnosis using MRI also indicate that future advances will not only rely on the architecture of the model but also on the quality of the data, uncertainty estimates, interpretability, imaging conditions, and validation of the model [2].

In medical AI, reliability and interpretability are as important as classification accuracy. Even highly accurate models may produce poorly calibrated probabilities; therefore, calibration is essential before such models can be considered reliable for clinical decision support. Assessing the meaningfulness of the predicted probabilities can thus be gained by calibration assessment based on measures like expected calibration error and negative log-likelihood. Likewise, visual explanation techniques like Grad-CAM can be used to investigate whether the model focuses on plausible anatomical areas or on artifacts in the image, borders, or specifics in the preprocessing. These factors are especially important for rare neurological diseases, where clinicians need transparent and uncertainty-aware decision support beyond just a predicted label [6,7].

To address these challenges, we propose RareNeuroXNet, a frequency-aware multi-branch attention framework for image-level classification of rare neurological diseases from brain MRI. The model fuses three complementary representations: a global branch for high-level anatomical context for the entire image, a local branch for high-precision feature extraction for local regions in the image, and a lightweight frequency-domain-based branch on the magnitude information from the Fast Fourier Transform. Attention-guided feature learning is then applied to these representations to further improve them before they are fused for final classification. The design is intended to capture complementary disease-related information that may not be fully represented by single-stream spatial models.

The first research question underlying this study is whether a multi-branch deep learning framework can enhance the image-level classification of rare neurological diseases under conditions of limited sample availability, heterogeneous imaging, and subtle structural variation. This study explores the effects of integrating global anatomical information with local fine-grained cues and frequency domain-based texture features on discrimination and probability reliability. Thus, in addition to accuracy-based metrics, cross-validation stability, calibration analysis, ablation experiments, internal baseline comparison, MCND cross-dataset benchmarking, and Grad-CAM-based visual explanation are also included in the evaluation.

This study also includes the MCND dataset as a complementary cross-dataset neurological MRI benchmark. The MCND consists of 16,400 brain MRI images from 8 classes: Alzheimer’s disease (stages), brain tumor (subtypes), multiple sclerosis, and normal controls. Since MCND is not a set of five rare neurological diseases used in the primary experiment, it is not considered same-task external validation. Instead, it is employed as an extra benchmark to investigate the model behavior for another neurological MRI classification task and to give a wider context for comparison with baseline architecture RareNeuroXNet.

The main contributions of this study are summarized as follows:

- This study addresses the underexplored problem of rare neurological disease classification from brain MRI, a topic that has received less attention than common MRI classification tasks such as brain tumor and Alzheimer’s disease classification.
- It proposes RareNeuroXNet, a frequency-aware multi-branch attention framework that integrates global anatomical representation, local fine-grained feature extraction, and FFT-based frequency-domain texture modeling.
- It evaluates the proposed framework using a comprehensive experimental protocol that includes classification metrics, calibration analysis, ablation experiments, and Grad-CAM-based visual explanation.
- It compares RareNeuroXNet with CNN- and transformer-based baseline models and includes an additional MCND cross-dataset benchmark to assess model behavior on a different neurological MRI classification task.
- It provides a cautious interpretation of the findings as image-level benchmark evidence and discusses key limitations related to curated balanced datasets, patient-level metadata, external validation, and 2D MRI analysis.

## 2. Literature Review

As deep learning can learn discriminative imaging features directly from data, it has emerged as one of the most important research fields for MRI-based neurological disease classification. The initial research was based on transfer learning with pre-trained CNN-based models like ResNet, DenseNet, VGG, and EfficientNet. The models showed that CNN feature extraction can perform well in brain MRI classification, especially in the cases of limited labeled medical data. Transfer learning proved particularly useful, as it is able to cut down the need for training large models from scratch and offered a practical approach for computer-aided diagnosis in brain tumor and neurodegenerative disease applications [3,6,8,9,10].

Although conventional single-branch CNN models are successful, there are still several limitations in neurological MRI analysis. Brain disorders may be very similar within classes, have subtle anatomical variation, and have different imaging appearances. Consequently, models that rely solely on spatial properties across the entire image might fail to capture the fine-grained variations in textures or patterns of interest in the disease. These limitations were addressed by subsequent work that used image enhancement, wavelet-based image preprocessing, multi-stage pipelines, ensemble learning, and multi-branch feature extraction. It was demonstrated that these methods enabled a more accurate classification than using a single image stream alone, particularly for brain MRI classification problems with multiple classes [9,10,11,12].

In recent years, models based on attention (attention-based models) and transformers (transformer-based models) have gained more and more attention in medical image classification. CNNs perform well in capturing local texture, edge, and anatomical details, while transformer modules excel in capturing long-range dependencies and global contextual relationships. Therefore, hybrid CNN–transformer models, cross-attention fusion strategies, self-attention mechanisms, and Swin Transformer-based architectures have been taken into consideration for MRI classification tasks. The results of these studies suggest that the attention mechanisms can enhance feature selectivity and could facilitate focusing on the more informative spatial regions or feature channels of the model [13,14,15,16,17,18,19].

Studies in recent years, however, have stressed that complex architecture is not enough for reliable performance. The importance of dataset quality, the consistency of annotations, augmentation strategy, modality selection, calibration, interpretability, and external validation continues to be paramount for medical AI evaluation. High accuracy is often reported in studies based on deep learning and MRI that are performed on curated datasets, but when tested in other scanners, institutions, imaging protocols, and patient cohorts, performance can fall significantly short. This is especially relevant for rare neurological disease classification, where datasets tend to be small and balanced for research and may not have patient-level metadata [2,20,21,22,23,24,25].

Neurological diseases, which are rare for most modeling studies of deep learning, are still underrepresented. A scoping review of deep learning for rare diseases revealed that CNN-based models are frequently used in rare-disease applications, although there is some variation in research across disease groups. This is because rare disease applications in the field of neurology are less developed compared to many other rare disease applications in the field of neoplastic disease and genetic disease, in part due to limited access to well-annotated imaging cohorts, variable phenotypes, and limited data [6]. The difficulties these challenges pose point to the need for models that are capable of learning from small data sets and that offer careful considerations of reliability, calibration, and interpretability.

Robustness-oriented modeling and generalization-awareness modeling are also emphasized in recent works. Some methods have been suggested to enhance the classification performance and generalization, such as transformer-only architectures, multi-scale networks, multi-channel MRI models, diffusion-assisted augmentation, and ensemble methods. These techniques indicate that complementary representations may prove helpful if patterns relevant to the disease are present at multiple spatial scales or in different feature spaces. This observation is closely related to the motivation of RareNeuroXNet for encoding global anatomical context, local fine-grained representation, and frequency domain texture information into a single framework [2,26,27,28,29].

The classification of Alzheimer’s disease and brain tumors follows a similar trend. The extension of earlier CNN-based models with transformer modules, stage-wise classification, ensemble learning, and multi-scale feature extraction is a trend that has been growing. Because these developments are applicable to rare neurological MRI classification, they highlight the necessity of local disease cues and structural context. However, many of these studies are still confined to the common neurological disorders or tumor databases, and they do not directly apply to the classification of rare neurological disorders without task-specific evaluation [30,31,32,33,34].

Another frontier is the application of large-scale foundation models, based on brain MRI images, which have been previously trained. These approaches attempt to discover generalizable representations in large unlabeled or weakly labeled datasets and then fine-tune them for downstream clinical applications. For rare neurological diseases, labeled information may be particularly useful, as it is difficult to obtain. These models, however, still need to be carefully validated, calibrated, assessed, and analyzed in terms of interpretability before they can be trusted in a clinical setting. Hence, the current study also treats RareNeuroXNet as a task-specific image-level benchmark framework and emphasizes that it is essential to compare it with other foundation-model and transformer-based models soon [20,32,35].

Overall, the literature indicates a shift in neurological disease classification from the classical CNN transfer learning to hybrid systems, attention-based systems, transformer-based systems, and calibration-aware systems. Most studies, however, continue to be based on the most common diseases, including brain tumors and Alzheimer’s. The study of rare neuro disorders has been less explored, especially in the aspects of calibration, ablation analysis, visual explainability, and cross-dataset benchmarking. This encourages the proposed RareNeuroXNet framework that incorporates global, local, and FFT-based frequency domain representations and assesses the model through classification metrics, calibration analysis, ablative experiments, visual explanations using Grad-CAM analysis, baseline comparison, and a further MCND cross-dataset benchmark.

The representative MRI-based neurological disease classification studies in Table 1 show the methodological evolution from traditional CNN transfer learning to hybrid attention and transformer-based and ensemble models. The reviewed studies demonstrate high accuracy in various brain MRI classification tasks, especially in the brain tumor and Alzheimer’s disease datasets. The majority of these, however, are assessed on common-disease data sets, not on rare neurological disease data sets, and therefore do not have direct relevance to the issue studied here. Further, the latter part of the table reveals that, while high accuracy is often reported for curated MRI datasets, many studies are limited in their evaluation by focusing only on tumor assessment, have limited external validation, have insufficient attention to calibration and interpretability, or are not specific to rare diseases. These drawbacks highlight the importance of a framework like RareNeuroXNet for rare neurological MRI classification tasks, which are assessed via both classification metrics and ablation analysis, calibration assessment, Grad-CAM explanation, and cross-dataset benchmarking.

## 3. Proposed RareNeuroXNet Model

**Figure 1:**
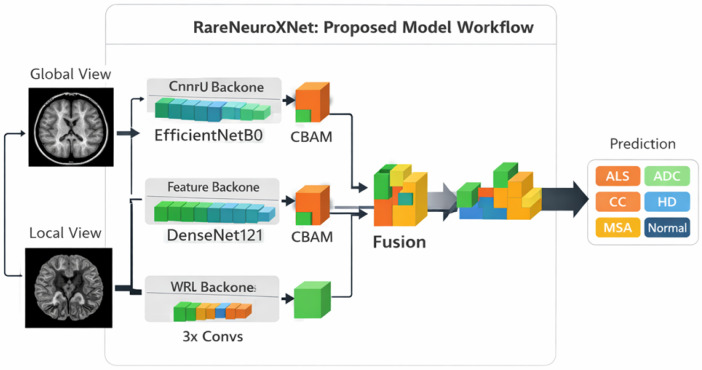
RareNeuroXNet Proposed Model.

The proposed RareNeuroXNet architecture for image-level classification of rare neurological diseases from brain MRI is shown in Figure 1. The model learns complementary disease representations by fusing three views of the same MRI slice: global anatomical, local fine-grained, and frequency-domain representations. The motivation for this design is the heterogeneity of the appearance of rare neurological diseases, in which some abnormalities could be seen as a generalized structural change, while others could be local or texture dependent. RareNeuroXNet aims to fuse whole-image context, regional anatomical information, and FFT-based spectral cues in an attention-guided manner.

RareNeuroXNet has a total of six stages: input preparation, global branch feature extraction, local branch feature extraction, frequency branch modeling, attention-guided feature fusion, and final classification. To begin with, every MRI image is processed and transformed into various complementary representations. Second, discriminative features are extracted by the pretrained deep backbones in the global and local views of the image. Third, frequency-domain information that is related to texture variation and structural variation is modeled using an FFT-based branch. Fourth, attention modules enhance spatial and channel-wise significance of the extracted features. Fifth, the learned representations are combined into a single embedding. Lastly, the fully connected layers produce the probability of distribution of the target rare classes of neurological diseases.

RareNeuroXNet has several methodological benefits. First, it is a combination of global and local MRI images, which allows the simultaneous learning of both the large context and local cues of disease. Second, it adds a frequency-sensitive branch, which adds spectral content that is frequently ignored by traditional image models. Third, it uses attention-guided refinement to highlight more discriminative responses. Fourth, it facilitates ablation analysis since it enables selective ablation of local, FFT, or attention components. Finally, it is also computationally manageable and more expressive than traditional single-backbone classifiers. These properties make RareNeuroXNet suitable for controlled image-level benchmarking of rare neurological disease classification, where visual patterns may be subtle and heterogeneous.

The study was designed to evaluate RareNeuroXNet as an image-level benchmark system for rare neurological disease classification. The design includes three complementary components: a task-specific multi-branch architecture, an internal stratified cross-validation protocol, and a reliability assessment using calibration metrics and visual explanation. A multi-branch architecture was chosen to evaluate if the global anatomical context, local fine-grained features, and FFT-based frequency information give complementary representations in the context of rare neurological MRI classification. The validation protocol was designed to evaluate internal stability by stratified cross-validation and include an independent hold-out test set for final evaluation. In addition to classification accuracy, the evaluation includes ablation analysis, baseline comparison, calibration metrics, and Grad-CAM visualization to assess component contribution, probability reliability, and qualitative model behavior. So the research design could be considered appropriate for an initial controlled image-level benchmark study, and patient-level validation and external testing on the same tasks would be required to generalize the clinical context.

### 3.1. Input Preparation

RareNeuroXNet receives a two-dimensional brain MRI image that is resized to a fixed spatial resolution. The model creates three complementary representations of each image to boost the diversity of features. The first representation is the global view, which maintains the entire MRI slice and gives the whole image an anatomical context. This view is designed to capture large-scale anatomical structure and global disease-related patterns.

The second representation is the local view, which is created by first cropping to the center and then resizing. This view restricts the model’s focus to central brain regions that may contain subtle disease-specific cues. The third representation is the frequency-domain view, which can be obtained by calculating the FFT magnitude image. This perspective can include aspects like texture, edge concentration, and patterns of intensity distribution that might not be captured well in the spatial domain. RareNeuroXNet aims to assess if global, local, and frequency-domain information are complementary for rare neurological disease classification through this combination of three representations.

### 3.2. Global Branch Feature Extraction

EfficientNetB0 was selected for the global branch because it provides a favorable balance between representational capacity and computational efficiency. The compound scaling strategy is designed to uniformly scale the network’s depth, width, and input resolution, enabling the network to learn informative features while keeping the model complexity relatively low. This feature is especially helpful if the medical imaging datasets are small to medium-sized, as very large backbones can lead to overfitting. EfficientNetB0 is used in RareNeuroXNet to process the entire MRI slice and to learn the whole-brain structural patterns and global anatomical organization as well as large-scale disease-related variations. Thus, the global branch provides broad contextual information that complements the fine-grained local and frequency-domain representations learned by the other branches.

An attention module is used to further refine the output feature maps of the global branch before global average pooling. This refinement that guides attention enables the model to focus on the most informative channels and spatial regions and to inhibit less informative responses. Therefore, a more discriminative whole image representation can be achieved without significant increase in computational complexity in the global branch.

### 3.3. Local Branch Feature Extraction

DenseNet121 was selected for the local branch because its dense connectivity promotes feature reuse and improves gradient flow across layers. These properties are helpful in fine-grained MRI classification because the texture changes, small structural variations, and abnormalities in certain regions may contain important diagnostic information. To this end, RareNeuroXNet provides the local branch with an MRI view with a center focus, such that the model is able to focus more on central brain structures and more salient cues in the local MRI that may not be as apparent in the full-slice representation. Thus, EfficientNetB0 on the global branch and DenseNet121 on the local branch are designed to complement each other in anatomical context and fine detail representation without redundancy.

The cropped MRI input is fed into a DenseNet121 backbone after removing its original classification head. The cropped MRI input is then processed by DenseNet121 without the original classification head. This pathway aims to identify structural patterns, textural differences, and subtle anatomical variations at a local level, which may contribute to classifying similar rare neurological disease classes that are visually similar. As in the global branch, an attention module is applied to the local feature map before pooling. This attention guided refinement suppressed less informative responses and focused more on the diagnostically relevant local structures to create a more discriminative region representation for final fusion.

### 3.4. Frequency-Aware Branch

The frequency-aware branch complements spatial-domain learning by extracting texture-sensitive spectral representations. CNN backbones are effective at extracting spatial features, but not necessarily explicit frequency-domain features. In MRI classification, frequency information can, however, reveal fine details in the distribution of intensities, edge concentration, and tissue texture that the original pixel domain might not be able to capture completely.

To construct this branch, the MRI input image is converted to the FFT magnitude analysis. A lightweight convolutional subnetwork of stacked convolutional and pooling layers is then used to process the resulting frequency map. This branch progressively transforms the spectral map into a compact frequency-aware representation. In contrast to the two pretrained branches, the FFT pathway is intentionally kept lightweight to limit model complexity while still contributing complementary spectral information. This architecture enables RareNeuroXNet to combine three different but closely related views of the same MRI image, namely contextual anatomy, localized pathology and frequency-based texture behavior.

### 3.5. Attention Module

The Convolutional Block Attention Module (CBAM) is employed in RareNeuroXNet to enhance the feature maps of the global branch and the local branch. CBAM has two consecutive stages: channel attention and spatial attention. The channel attention is an estimate of the relative importance of feature channels, and the spatial attention is an estimate of informative spatial regions within the feature maps.

This refinement is useful because pretrained CNN backbones may generate many feature responses, not all of which are equally relevant for classification. CBAM offers a reweighting of the feature channels and spatial locations, making the model focus more on informative responses and reduce less informative responses. CBAM is used after the global and local backbone but prior to feature pooling and fusion in the proposed architecture. The attention module, therefore, is a feature-refinement mechanism that enhances discriminability among representations without substantially increasing computational cost.

### 3.6. Fusion Strategy

After feature extraction and attention refinement, the outputs of the three branches are combined using a fusion module. Fusion aims to combine information acquired through the global, local, and frequency-sensitive pathways to obtain a single representation of this information. Having a single branch would mean that the model would not capture vital cues that are more apparent in a different representation. Therefore, the fusion stage is a central component of RareNeuroXNet because it integrates complementary anatomical, local, and spectral cues.

In the basic form of the model, the pooled features of the branches that are available are appended to create one composite feature vector. This is a simple yet efficient strategy of retaining all the learned information of the contributing pathways. In another form of ablation, weighted fusion is used, where the relative contribution of each branch is controlled by learnable weights. This enables the network to focus dynamically on the stream that is more informative to a particular disease pattern. The fused representation is then normalized and sent to the classifier head. The model uses structural, local, and spectral cues in a coordinated fashion through this process.

### 3.7. Classification Head

The final stage of RareNeuroXNet is the classification head, which predicts the disease probabilities on the input fused feature vector. The classifier is made up of two fully connected layers with nonlinear activation and dropout regularization and a softmax output layer. The fully connected layers transform the fused representation into a compact task-specific latent representation, while dropout reduces overfitting.

Each target class has one neuron in the output layer, and the softmax function transforms the logits into normalized probabilities of the classes. The class with the highest softmax probability is selected as the final prediction. This design facilitates the classification of multi-class rare neurological diseases in a simple and computationally efficient way.

Algorithm 1 presents the complete training algorithm of RareNeuroXNet. The algorithm is designed in line with the research design, where the input-view generation is split from the extraction of features related to the specific branch, the refinement of the attention mechanism, the fusion of features, the training of the classifier, and the fine-tuning. The local branch, FFT branch, CBAM module, and fusion strategy can also be selectively removed/modeled during ablation analysis, making this structure suitable for component-level evaluation. Hence, it offers a repeatable model to assess whether the overall image-level classification results come from different architectural parts.
**Algorithm 1** Training Procedure of RareNeuroXNet  1:**Input:** MRI training set $\left(D={\left\{\left(x_{i},y_{i}\right)\right\}}_{i=1}^{N}\right)$  2:**Output:** Trained RareNeuroXNet model (M)  3:Initialize the model configuration:  4:   Global branch backbone: EfficientNetB0  5:   Local branch backbone: DenseNet121  6:   Frequency branch: lightweight CNN  7:   Attention module: CBAM  8:   Fusion module: concatenation  9:For each MRI image $\left(x_{i}\right)$ in the dataset (D):10:   Resize the full image to obtain the global view $\left(x_{i}^{g}\right)$11:   Crop and resize the image to obtain the local view $\left(x_{i}^{l}\right)$12:   Compute the FFT magnitude image to obtain the frequency view $\left(x_{i}^{f}\right)$13:Feed $\left(x_{i}^{g}\right)$ into the global backbone to obtain the global feature map $\left(F_{i}^{g}\right)$14:Feed $\left(x_{i}^{l}\right)$ into the local backbone to obtain the local feature map $\left(F_{i}^{l}\right)$15:Feed $\left(x_{i}^{f}\right)$ into the frequency CNN to obtain the frequency feature map $\left(F_{i}^{f}\right)$16:Apply CBAM to the global and local feature maps:17:   $\left({\hat{F}}_{i}^{g}=CBAM\left(F_{i}^{g}\right)\right)$18:   $\left({\hat{F}}_{i}^{l}=CBAM\left(F_{i}^{l}\right)\right)$19:Apply global average pooling:20:   $\left(z_{i}^{g}=GAP\left({\hat{F}}_{i}^{g}\right)\right)$21:   $\left(z_{i}^{l}=GAP\left({\hat{F}}_{i}^{l}\right)\right)$22:   $\left(z_{i}^{f}=GAP\left(F_{i}^{f}\right)\right)$23:Fuse the branch representations:24:   Concatenation version: $\left(z_{i}=\left[z_{i}^{g};z_{i}^{l};z_{i}^{f}\right]\right)$25:   Weighted fusion version: $\left(z_{i}=Fusion\left(z_{i}^{g},z_{i}^{l},z_{i}^{f}\right)\right)$26:Pass the fused representation through fully connected layers with dropout:27:   $\left(h_{i}=FC_{2}\left(Dropout\left(FC_{1}\left(z_{i}\right)\right)\right)\right)$28:Compute class logits and softmax probabilities:29:   $\left(p_{i}=Softmax\left(h_{i}\right)\right)$30:Calculate the loss using categorical cross-entropy with label smoothing31:Update the model parameters using the Adam optimizer32:Freeze and train the classifier head in Stage 133:Partially unfreeze the upper backbone layers and fine-tune them in Stage 2 using a smaller learning rate34:Repeat the optimization process until the stopping criterion is satisfied35:Return the trained RareNeuroXNet model (M)

### 3.8. Implementation and Training Details

The MRI images were resized to 224 × 224 pixels and then fed into the model. Three complementary inputs were created for each image, one full global image, one local image cropped from the middle, and an FFT image of the frequency spectrum. A center crop ratio of 0.82 was used to create the local view, which was then resized to the same input resolution. This crop was used to highlight the brain’s central structures while minimizing background information. This frequency view was constructed by converting the image to grayscale, calculating a 2D Fast Fourier Transform, and moving the center 0 frequency component to the middle, taking the log of the FFT magnitude, normalizing it between [0, 1], and arranging the pixels into three channels. For the present implementation only the FFT magnitude was utilized; no phase information was used.

The CBAM was applied once the global and local backbone feature maps were applied. The channel attention block was composed of global average pooling with two dense layers and sigmoid activation for reweighting feature channels. In the spatial attention block, the average and the maximum projection were used along the channel dimension, and the two maps were concatenated before passing through a 7 × 7 convolution (with sigmoid activation) to produce the spatial attention map. This design enabled the model to enhance feature responses channel-wise and spatially before pooling and fusion.

The model was run by setting a fixed seed number (42) of the RNG. To ensure that training was conducted in two stages. In the first phase, the pretrained EfficientNetB0 and DenseNet121 backbones were frozen, and the new fusion and classification layers were trained. The top 20 layers of each pretrained backbone were unfrozen and fine-tuned with a smaller learning rate in the second stage. The classifier head was trained using the Adam optimizer with a learning rate of $1\times 10^{-3}$ and fine-tuned with $1\times 10^{-4}$. It was trained with a batch size of 16, categorical cross-entropy loss with label smoothing of 0.03, and dropout of 0.35 in the classification head.

The model has been trained for 6 epochs of training the classifier heads and 8 epochs of fine-tuning. The early stopping technique was used to monitor the validation accuracy, and the learning rate reduction was monitored based on the validation loss. Data augmentation was performed by flipping horizontally at random, rotating the images in the range of +10 to −10 degrees at random, and slightly changing the brightness/contrast of the images during the training. To make a fair comparison of architectural differences, the same training configuration was applied to the full model and ablation variants and compared to the baseline.

## 4. Experimental Results and Discussion

The results are shown in five parts. First, internal cross-validation metrics of classification, discrimination, and calibration are provided for RareNeuroXNet. Secondly, visualization is provided for fold-wise behavior using training curves, confusion matrix, ROC curves, PR curves, reliability diagrams, and confidence histograms. Third, the MCND experiment is described as a cross-dataset neurological MRI experiment, rather than as the same-task external validation. The contribution of each model component will be analyzed using ablation analysis as the fourth analysis. The model interpretability and comparative performance are evaluated through the use of Grad-CAM and baseline internal comparisons of the model with standard architectures.

Complete patient-level identifiers were not available, and it would not be possible to rule out exact patient-level leakage. Furthermore, the fact that there are some duplicate or near-duplicate MRI images in the partitions is still a drawback of the public dataset. Thus, the reported results are not intended as an independent patient-level diagnostic performance but are benchmark results only to be interpreted at the image level.

### 4.1. Dataset

This paper used the Rare Neurological Diseases MRI Curated Edition dataset, which is derived from a broader benchmark diagnostic MRI and medical imaging collection [36,37]. The curated subset contains five rare neurological disease categories: Fukuyama muscular dystrophy, Hallervorden–Spatz disease, Moyamoya disease, pachygyria with cerebellar hypoplasia, and Walker–Warburg syndrome. The dataset includes 2000 two-dimensional MRI images, with 400 images per class. The public release provides image-level samples rather than complete volumetric MRI examinations. Therefore, detailed acquisition metadata, including scanner vendor, field strength, institution, MRI sequence type, number of unique patients, number of slices per patient, and full clinical or radiological verification details, are not available. This limitation is important when interpreting the results because the study should be considered an image-level benchmark evaluation rather than a full clinical diagnostic validation.

The dataset was initially organized into training, validation, and test partitions using a 70/15/15 ratio, resulting in 1400 training images, 300 validation images, and 300 hold-out test images. Each disease class contributed 280 training images, 60 validation images, and 60 test images, as shown in Table 2. Balanced design supports controlled model training, fair class-wise evaluation, and interpretable ablation analysis. However, it does not reflect the natural prevalence distribution of rare neurological diseases in clinical practice, where substantial class imbalance is expected. Therefore, the reported macro-level performance should be interpreted as controlled benchmark performance on a balanced dataset rather than direct evidence of real-world diagnostic behavior.

**Figure 2:**
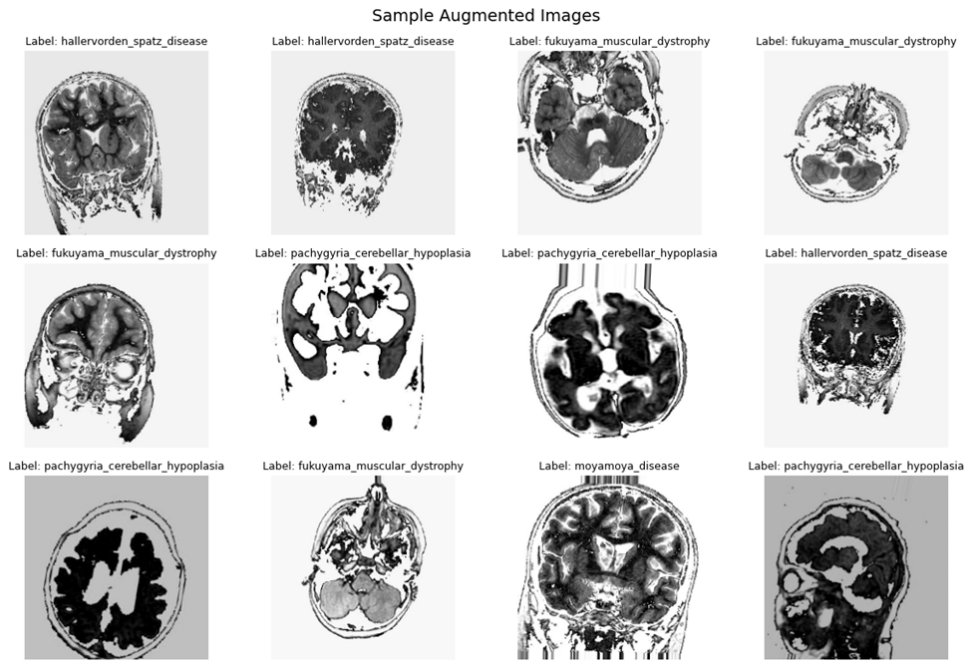
Dataset Sample Augmented images.

Figure 2 shows samples of MRI images from five classes: Fukuyama muscular dystrophy, Hallervorden–Spatz disease, Moyamoya disease, pachygyria with cerebellar hypoplasia, and Walker–Warburg syndrome.

### 4.2. Evaluation Protocol and Leakage Control

To separate internal validation from final testing, the original training and validation partitions were combined to form a development set of 1700 images. Five-fold stratified cross-validation was then applied only within this development set. In each fold, the model was trained on four development folds and validated on the remaining fold. The independent test set of 300 images was kept fixed and was not used during folding generation, model training, hyperparameter selection, or calibration fitting. This design avoids direct use of test images during cross-validation and preserves a hold-out set for final evaluation.

However, the public dataset does not provide complete patient-level or subject-level identifiers. Therefore, strict subject-level separation between training, validation, and test partitions could not be verified. This is an important limitation because multiple MRI slices or visually similar images from the same subject may be correlated, and their presence across different partitions could inflate performance estimates. Consequently, the reported results should be interpreted as image-level internal benchmark performance on the available curated dataset rather than definitive evidence of patient-level or clinical generalization. Future studies should use datasets with patient identifiers so that all slices from the same subject remain within a single fold or partition.

The design of this evaluation was chosen to ensure internal stability evaluation while holding back the final hold-out testing. To evaluate the model’s consistency when trained and validated with different subsets of the development set, five-fold cross-validation was performed. Five-fold cross-validation was conducted for consistency across different training/validation partitions, and the independent test set was used to assess the model. Apart from that, ablation analysis, baseline comparison, calibration metrics, Grad-CAM visualization, and MCND cross-dataset benchmarking were performed to assess the model on multiple fronts. Since no full patient-level identifiers were provided, however, the design should be seen as an image-level benchmark protocol and not the entire clinical validation protocol.

### 4.3. Complementary Cross-Dataset Neurological MRI Benchmark: MCND Dataset

To further evaluate the model beyond the primary rare neurological disease dataset, an additional cross-dataset experiment was conducted using the Multi-Class Neurological Disorder (MCND) brain MRI dataset. The MCND dataset is publicly available through Kaggle and includes MRI images from three neurological disorder groups: Alzheimer’s disease (AD), brain tumor (BT), and multiple sclerosis (MS). It contains 16,400 human brain MRI images categorized into eight classes: AD-MildDemented, AD-ModerateDemented, AD-VeryMildDemented, BT-glioma, BT-meningioma, BT-pituitary, MS, and Normal healthy controls.

Because the MCND dataset does not contain the same five rare neurological disease classes used in the primary experiment, it was not treated as same-task external validation. Instead, it was used as a complementary cross-dataset neurological MRI benchmark to assess model behavior on a different and more heterogeneous classification task. This benchmark was also used to compare RareNeuroXNet with standard baseline models, including CNN-based and transformer-based architectures, under the same experimental protocol. This design strengthens the comparative evaluation while maintaining a clear distinction between cross-dataset benchmarking and direct external validation for the target rare neurological disease classes.

### 4.4. Results

Table 3 shows the fold-wise cross-validation results for the main curated rare neurological disease set using RareNeuroXNet. The model has demonstrated high classification results across all the five folds, with the accuracy, macro precision, macro recall, macro F1 score, and weighted F1 score being in the range of 0.98 to 1.00. The best results were found in Folds 2 and 5, with most classification metrics at 1.00, and the lowest in Fold 3, with most classification metrics being around 0.98. Even with this slight decrease, however, the difference between folds was not large, meaning the model was consistent across the different partitions of the development set. The calibration results also showed a high level of reliability in the probability estimates, with very low expected calibration error (ECE) values ranging from 0.00 to 0.01 and negative log-likelihood (NLL) values ranging from 0.01 to 0.05. These values indicate that the model’s predictions were consistent with the actual results for most of the cases. The NLL value was highest in Fold 3, but still low, and was consistent with the slightly lower classification performance in Fold 3.

Hence, the cross-validation outcomes show that RareNeuroXNet performed well in terms of discriminating the classes and calibrating it on all fivefold. Internal benchmark results, however, need to be treated with caution, as they are curated, balanced, and evaluated at the image level, and there are no complete patient-level identifiers. Thus, the subject-level independence could not be fully verified, and correlated images, duplicate or near-duplicate samples, or subject overlap between folds cannot be completely ruled out. Therefore, good classification and calibration performance near the tops of the current curated benchmark should be taken as an indicator that high image-level separability exists within the curated benchmark and not necessarily as proof of patient-level or clinical generalization.

Table 4 shows the cross-validation performance measures’ mean and standard deviation of RareNeuroXNet. The model had good and stable classification accuracy with a mean accuracy of 0.9924 ± 0.0061. Macro F1-score and weighted F1-score were also very high at 0.9924 ± 0.0061, showing almost balanced performance across the different classes and that the model was not heavily relying on majority class predictions. This low standard deviation also demonstrates consistency across the five folds of cross-validation in the model’s performance. The discrimination scores were also extremely high, with a macro one-vs-rest ROC-AUC of 0.9998 ± 0.0002 and a macro-PR-AUC of 0.9992 ± 0.0007 in the considered benchmark, showing high class separability. Furthermore, the calibration metrics exhibited good reliability with ECE = 0.0052 ± 0.0029 and NLL = 0.0276 ± 0.0159, meaning the predicted probabilities were well calibrated within the image-level benchmark class labels observed. The variability was slightly larger in NLL than in ECE, but these were very low, and this confirmed the probabilistic reliability of the model. The results of these strong findings should be interpreted with caution, as the data set is curated, balanced, and evaluated at the image level, and full patient identifiers are not available. So, it could not be fully confirmed that all the subjects were independent of each other, and there is a possibility of duplicate or near-duplicate images, correlated slices, or subject overlap across partitions. The reported scores must be interpreted, therefore, as evidence of good performance against the internal benchmark within the current data set, not as evidence of patient level or clinical generalization.

**Figure 3:**
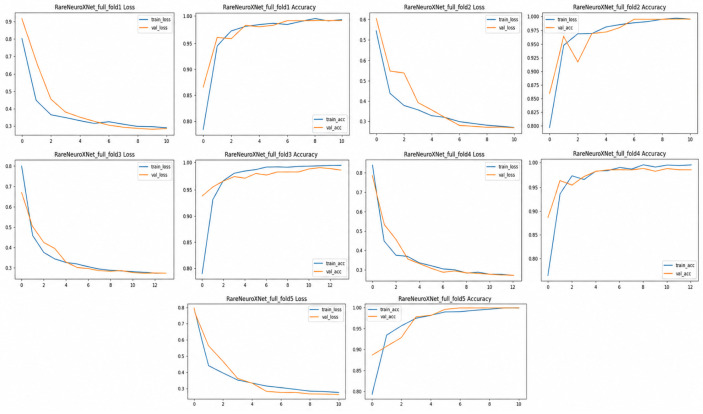
Accuracy and loss curves for cross-validation of the proposed model.

The training and validation curves with accuracy/loss are shown for RareNeuroXNet across the five folds of cross-validation in Figure 3. In general, the loss curves gradually decrease with each epoch of training, and the accuracy curves quickly improve in the initial epochs and converge toward high accuracy, indicating that the model is optimized well and achieves consistent convergence across the folds. In most folds, the training curve does not differ drastically from the validation curve, indicating consistent learning behavior that shows no pronounced difference between the training and validation performance. But the curves should be interpreted with caution, as it is possible that the curve is overfitting, learning something from the patients alone, or that it is influenced by correlated-image effects, as complete patient-level identifiers are not available. The convergence of Fold2 appears to be particularly rapid, and the convergence of the other folds also occurs in a smooth manner with slight fluctuations at the epoch level, indicating the stability of the training procedure within the evaluated image-level benchmark.

**Figure 4:**
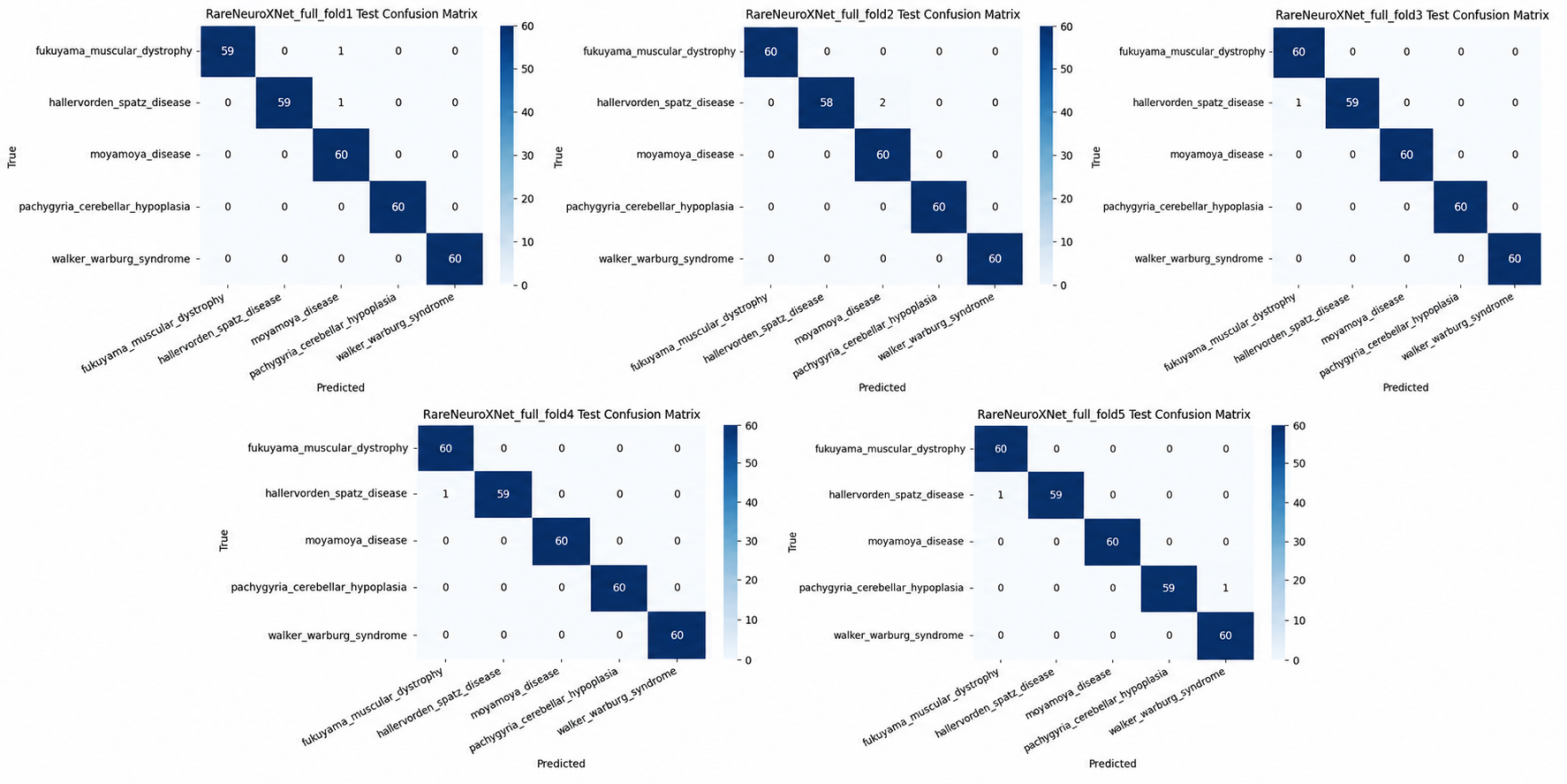
Confusion matrices for cross validation of the proposed approach.

The confusion matrices of RareNeuroXNet are shown in Figure 4 for the five cross-validation folds. Accordingly, most of the predictions were focused on the main diagonal of the matrices, meaning that the model could correctly classify most samples from the five rare neurological disease classes: Fukuyama muscular dystrophy, Hallervorden-Spatz disease, Moyamoya disease, pachygyria with cerebellar hypoplasia, and Walker-Warburg syndrome. The off-diagonal errors along the folds are limited in number and are primarily the confusion between similar-appearing disease classes (Hallervorden–Spatz disease and Fukuyama muscular dystrophy) or sometimes between different disease classes (Moyamoya disease and Walker–Warburg syndrome). The overall confusion matrix presents a picture of good internal discriminative performance of the model on the curated image-level benchmark, which, however, should be interpreted with caution due to the lack of external generalization and patient-level independence.

**Figure 5:**
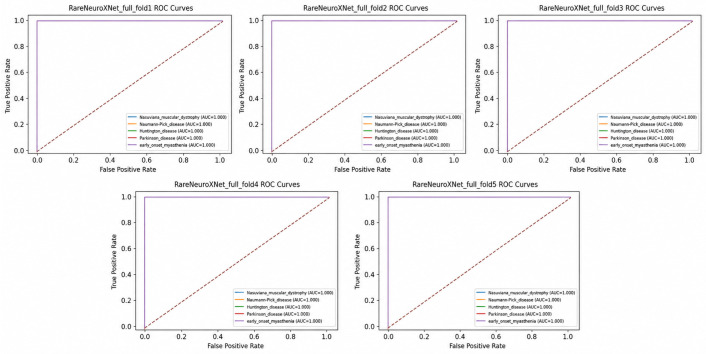
ROC curve for cross-validation of the proposed model.

The ROC curves of RareNeuroXNet on the 5 cross-validation folds are plotted in Figure 5. The curves of the five rare neurological disease classes are located near the top left of the plots, which suggests that the discrimination of each class from the rest of evaluated classes in the benchmark is good. The class-wise AUC values are nearly 1.00 across the folds, indicating that the models learned display a good separability between the selected categories of diseases. The fold-wise variation is small, and the shape of the ROC is close to an ideal shape in most of the folds. The model shows good internal discriminative performance with these results; however, the near-perfect ROC performance should not be viewed as the model’s ability to generalize its performance at the patient or external level without further validation.

**Figure 6:**
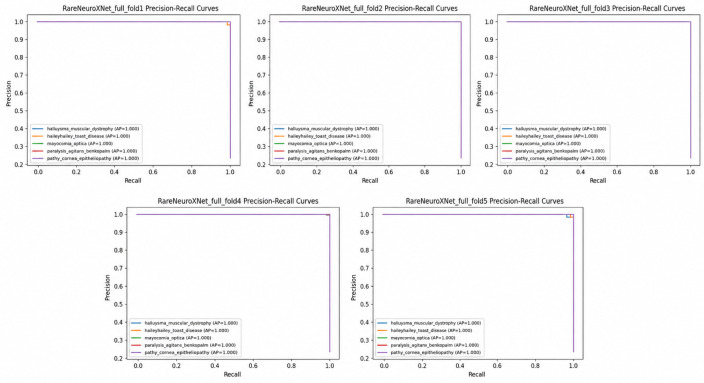
AUPR Curve for cross-validation of the proposed model.

We report the precision–recall curves of RareNeuroXNet in the five cross-validation folds in Figure 6. Curves of the five rare neurological disease classes are grouped around the upper right corner of the plots, meaning high precision and recall in the image-level test benchmark. Average precision scores are nearly 1.00 for all folds, indicating good positive predictive value for the selected classes. The fold-wise PR curves are, however, very similar, thus confirming the internal stability of the learned representations. Like the ROC results, the near-perfect pattern of PRs should be used with caution, as the dataset is curated and balanced, and independence of patients and external validation using the same tasks are required to assess generalizability.

**Figure 7:**
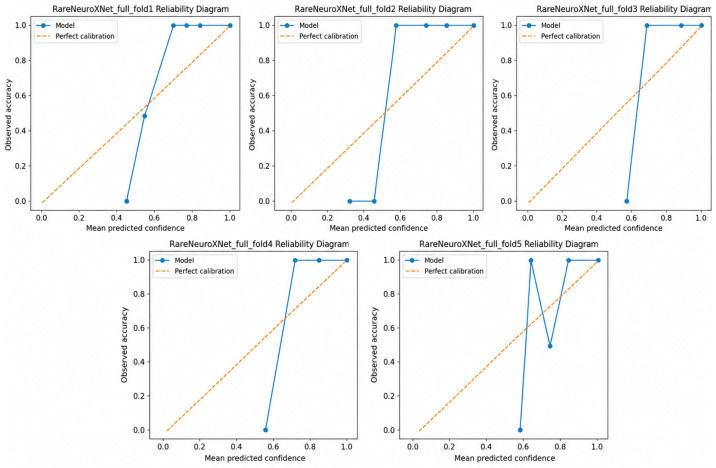
Reliability diagram for cross-validation of the proposed model.

The reliability diagrams of RareNeuroXNet are displayed for the 5 folds of cross-validation in Figure 7. These plots are graphs of the degree of confidence that the model gives versus the accuracy of that model; the ideal calibration is represented by a diagonal line. The overall shape of the curves is not that far off from the reference line, especially within the regions of high confidence, suggesting that the probabilities predicted by the curves are generally in line with actual results. There is a minor deviation in some folds, particularly in the mid-confidence ranges, but the pattern of calibration is consistent with the low ECE values found in the quantitative results. The results indicate that the probability calibration at the image level is good within the benchmark set for the current study but may need to be recalibrated using independent patient-level and external datasets, as confidence intervals can shift in different domains.

**Figure 8:**
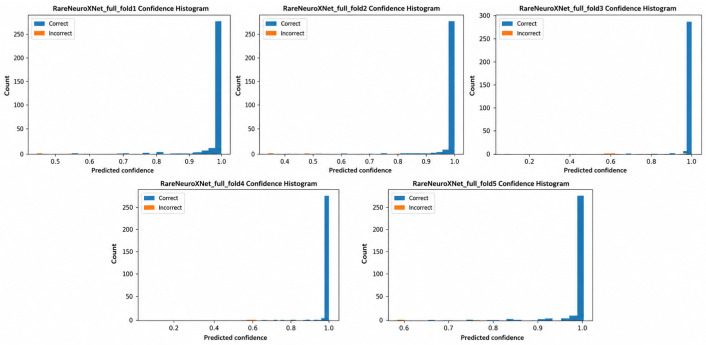
Confidence histogram for cross-validation of the proposed model.

The distribution of prediction confidence for correct and incorrect classifications is shown in the confidence histograms of RareNeuroXNet across the five folds of the cross-validation, presented in Figure 8. In most folds, most of the predictions fall within only a small portion of the high-confidence range, and these predictions are predominantly correct, suggesting high model confidence in correct classifications in the assessed benchmark. There are not many wrong predictions in the output, and they fall randomly in the lower or middle confidence intervals. The reliability diagrams and low calibration errors are consistent with this pattern and imply a good level of confidence behavior on the curated image-level dataset. Calibration and confidence reliability should be tested on independent external data sets but may vary depending on imaging conditions and patient population and should be evaluated accordingly.

**Figure 9:**
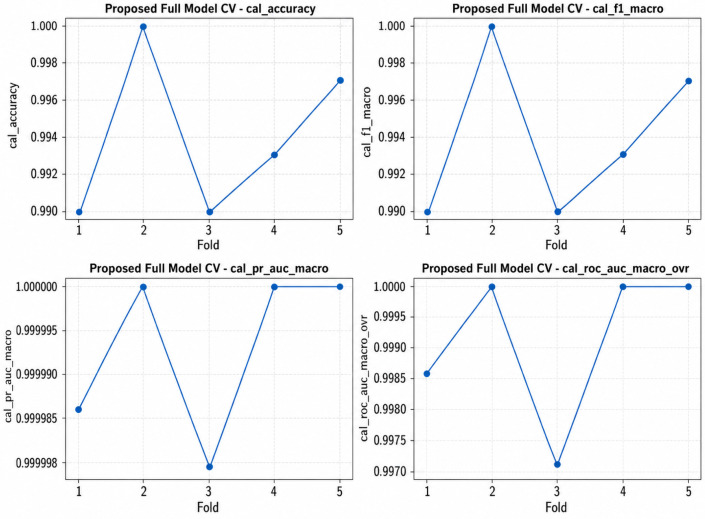
Metrics of proposed model cross-validation.

The cross-validation results are summarized in Figure 9, which shows the performance of RareNeuroXNet based on four different calibration metrics: accuracy, macro F1 score, macro-AU PR, and macro-AU ROC. The calibrated accuracy and macro F1-score are consistently high across all folds with only slight fluctuations, suggesting consistent internal performance of the evaluated image-level benchmark. The AUPR and AUROC curves are also relatively flat across the folds, indicating good class separability and precision–recall performance of the selected rare neurological disease classes. Fold 2 has the highest values, and the other folds have similar high performance. These results provide evidence of the internal stability of RareNeuroXNet over all cross-validation splits, but the results should not be interpreted as proof of wide clinical generalization without patient-level separation and independent external validation.

#### MCND Cross-Dataset Benchmark Results

**Figure 10:**
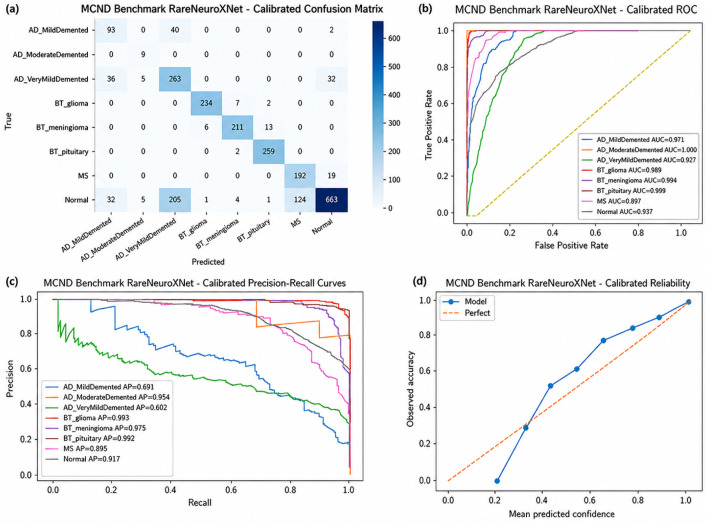
MCND benchmark performance of RareNeuroXNet.

The results of the calibrated performance of RareNeuroXNet on the MCND cross-dataset neurological MRI benchmark are shown in Figure 10. The eight MCND classes are AD-mild demented, AD-moderate demented, AD-very mild demented, BT-glioma, BT-meningioma, BT-pituitary, MS, and normal; and the corresponding confusion matrix is shown in Panel (a). The one-vs.-rest ROC curves are plotted in (b), the precision–recall curves in (c), and the reliability diagram in (d), which shows the agreement between the predicted confidence and the actual accuracy. These plots show classification behavior, class separability, PP (positive predictive) performance, and calibration quality for an alternative neurological MRI classification task. The performance of RareNeuroXNet is discriminatory in several classes with high ROC-AUC and average precision values as exhibited in the MCND benchmark, especially for the brain tumor classes BT-glioma, BT-meningioma, and BT-pituitary. A high diagonal performance is also observed in the confusion matrix for these classes of tumors and for the normal class. There is, however, some confusion that can be observed in the Alzheimer’s classes, with lower precision-recall scores for certain classes of AD, particularly in the AD-MildDemented, AD-VeryMildDemented, and Normal classes. This means that the performance of the model is different for the different disease groups and that classes that differ visually and clinically continue to be a challenge. The results do not constitute same-task external validation, as MCND does not include the five rare neurological disease classes of the primary experiment.

**Figure 11:**
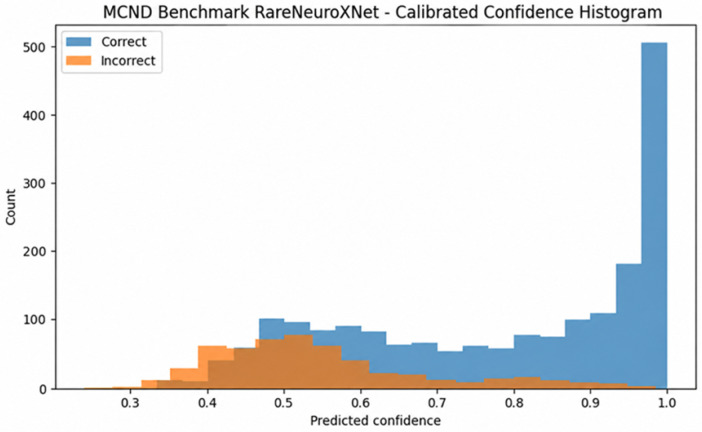
Calibrated Confidence Histogram for MCND dataset.

The calibrated confidence histogram of RareNeuroXNet on the MCND dataset is shown in Figure 11, where the calibrated confidence distribution of correct predictions is compared with the distribution of the incorrect predictions. The majority of correct predictions are clustered in the high prediction confidence level, particularly near the high confidence level values, suggesting that most of the model’s high prediction confidence levels are assigned to the correctly classified MCND samples. Those predictions that are wrong, on the other hand, tend to be more concentrated in the lower-to-mid level of the range, especially at the 0.4–0.6 level, which indicates greater uncertainty for many misclassified observations. This pattern suggests good confidence behavior, as the model is typically more confident in the correct prediction and less confident in errors. Some of these mispredictions, however, at relatively high confidence levels indicate that calibration was not perfect and that overconfident errors are still possible in cross-dataset settings that are heterogeneous. Thus, the confidence behavior needs to be reevaluated in independent rare neurological disease cohorts of the same task type prior to clinical application.

### 4.5. Ablation Study

Table 5 shows the ablation results of RareNeuroXNet after performing ablation on the original model and four variants, which are noFFT, noCBAM, weightedFusion, and noLocal, respectively. All the above values were excellent in the full model, in which accuracy, precision, recall, and F1-score were 0.99 with ROC-AUC and PR-AUC values of 1.00 and also having favorable calibration with ECE of 0.01 and NLL of 0.03. The test results showed that the full RareNeuroXNet configuration achieves the highest classification accuracy, discriminative power, and probabilistic confidence. The weightedFusion variant obtained slightly lower but still high accuracy, precision, recall, and F1-score values of 0.98, with the ROC-AUC and PR-AUC values of 1.00 demonstrating that other fusion strategies might also be effective to fuse the branch-specific representations. The accuracy and F1-score decreased to 0.97 and 0.96, respectively, when both the FFT branch and the CBAM module were removed, and both variants showed ROC-AUC and PR-AUC values near 1.00, suggesting that the model resulted in good class separation even without these auxiliary components. Thus, FFT and CBAM seem to offer valuable refinement and incremental performance benefit over and above being the only boosters for model performance. Conversely, when the local branch was removed, the noLocal variant had a significant drop in accuracy, precision, recall, F1-score, ROC-AUC, and PR-AUC (0.35, 0.38, 0.35, 0.31, 0.73, and 0.43, respectively). The calibration metrics also became very poor; ECE went up to 0.06 and NLL to 1.43 with unreliable prediction confidence in the absence of local discriminative features. The overall ablation tells that all components help the final model, with the biggest impact being the local branch for the performance and the supportive refinements being the FFT and CBAM. But the improvement is not significantly different between the full, noFFT, noCBAM, and weightedFusion variants, so these improvements should be interpreted cautiously and confirmed through additional fold-wise statistical comparisons and larger independent datasets.

#### 4.5.1. Fold-Wise Statistical Comparison of Ablation Variants

Table 6 provides a summary of the mean and standard deviation for the fivefold cross-validation results of the full RareNeuroXNet model, its ablation variants, and the DenseNet121 baseline (local-only model). The full model of the RareNeuroXNet demonstrated the highest image-level benchmark accuracy (0.9924 ± 0.0061) and macro-F1 score (0.9924 ± 0.0061), showing high classification performance and stable performance across folds. It was also the best-performing model in terms of discrimination, with a macro ROC-AUC score of 0.9998 ± 0.0002 and a macro PR-AUC score of 0.9992 ± 0.0007. Ablation variants and the baseline model performed poorly in terms of calibration, achieving ECE of 0.0074 ± 0.0013 and 0.0079 ± 0.0023, respectively, and NLL of 0.0428 ± 0.0313 and 0.0466 ± 0.0228, respectively, indicating that they made poorer predictions of the true probabilities associated with the underlying image-level labels.

Based on the results and the ablation, it can be seen that the local branch mainly affects the performance of the model. The removal of the local branch resulted in the most significant loss of accuracy (0.3412), macro-F1 (0.2779), ROC-AUC (0.6665), and PR-AUC (0.3610). The noLocal variant also exhibited the poorest calibration and ranked as the variant with the highest ECE (0.1077) and NLL (1.5574). The results indicate that the most discriminative information is contained in the evaluated benchmark in the fine-grained local MRI features. On the other hand, the removal of the FFT branch resulted in a less significant performance drop, with the noFFT variant obtaining a higher accuracy of 0.9206 and a higher macro-F1 of 0.9200. Likewise, the removal of CBAM had a more definite effect on performance than the removal of FFT, particularly in calibration, where ECE has deteriorated to 0.0661 and NLL to 0.3471. Thus, FFT and CBAM are described here as components of refinement in support of, but not on their own contributing to, the dominant role of the standard. The overall performance of the model was evaluated using DenseNet121 as the local-only baseline, which showed that the complex multi-view model outperforms the DenseNet121 model in terms of classification, discrimination, and calibration performance, substantiating the benefits of the larger multi-view model while affirming the most significant contribution made by the local branch.

Table 7 reports the five-fold cross-validation results of the complete RareNeuroXNet model, its ablation variants, and the DenseNet121 local-only baseline. The full model achieved consistently high image-level performance in all folds, with accuracy between 0.9853 and 1.0000 and macro-F1 between 0.9851 and 1.0000. It also exhibited very high discrimination scores with ROC-AUC and PR-AUC of 0.9998 and 0.9992, respectively, and low calibration errors across folds with ECE between 0.0000 and 0.0100 and NLL between 0.0100 and 0.0500.

The performance drop of the ablation variants was found to be different from the full model. The relatively competitive classification and discrimination ability of the noFFT variant showed that the loss of the frequency branch led to a slight (but not significant) degradation. The noCBAM variant demonstrated a more pronounced decrease in accuracy, macro-F1, PR-AUC, and calibration quality, indicating that attention-based feature refinement helps to create model stability and quality. The results of the weighted fusion variant were also competitive, although it was inferior to the full model in most folds. The noLocal variant had the largest loss of accuracy (0.2794–0.3794) and macro-F1 (0.2100–0.3440), with significantly lower ROC-AUC and PR-AUC values and significantly higher NLL values. It affirms the local branch has the highest predictive power in terms of the model’s discriminative power. The classification, discrimination, and calibration performance of the full RareNeuroXNet model showed statistically significant improvement for all folds compared with the DenseNet121 local-only baseline model. In general, the results obtained by the fold-wise approach support the effectiveness of the multi-view design and suggest that the principal performance contributor is the local branch and that FFT and CBAM are primarily providing refining support within the examined image-level benchmark.

The paired fold-wise statistical comparison of the full model RareNeuroXNet with each ablation variant is shown in Table 8 for the five-fold cross-validation setup. The mean difference was obtained by subtracting the corresponding variant from the full model. As such, positive scores for Accuracy and F1-macro suggest that the complete model performs better at classification; negative scores for ECE and NLL suggest that the complete model performs better at calibration, as lower scores are desired for these measures. The paired *t*-test results indicate that the full RareNeuroXNet model significantly outperforms all variants of the ablation model, as well as the local-only baseline model DenseNet121, for the classification and calibration metrics reported. The full model outperformed noFFT in terms of accuracy and F1-macro and outperformed noFFT and the model without the FFT branch in terms of ECE and NLL, respectively, demonstrating useful, complementary frequency-domain information from the FFT branch. Likewise, for no CBAM, the results were significantly better in the performance and calibration metrics, reflecting the effectiveness of attention-based feature refinement in CBAM. The full model also outperformed the model with weighted fusion, as this experiment demonstrated that the fusion strategy used achieved a better overall balance between classification performance and calibration. The largest differences were noted for the noLocal variant, where the local branch was removed, resulting in significant reductions of accuracy and F1-macro and significant losses of calibration. This validates that the local branch is the major contributor to RareNeuroXNet performance. The full model, compared to DenseNet121 local-only, also achieved significant gains in accuracy, F1-macro, ECE, and NLL, demonstrating that the wider multi-view design is beneficial beyond a single local backbone. The Wilcoxon *p*-values were still at 0.0625, however, which is likely due to the few folds included; statistical results must be interpreted with caution and are considered supportive rather than definitive evidence of patient-level clinical superiority.

#### 4.5.2. Grad-CAM Visual Explainability

**Figure 12:**
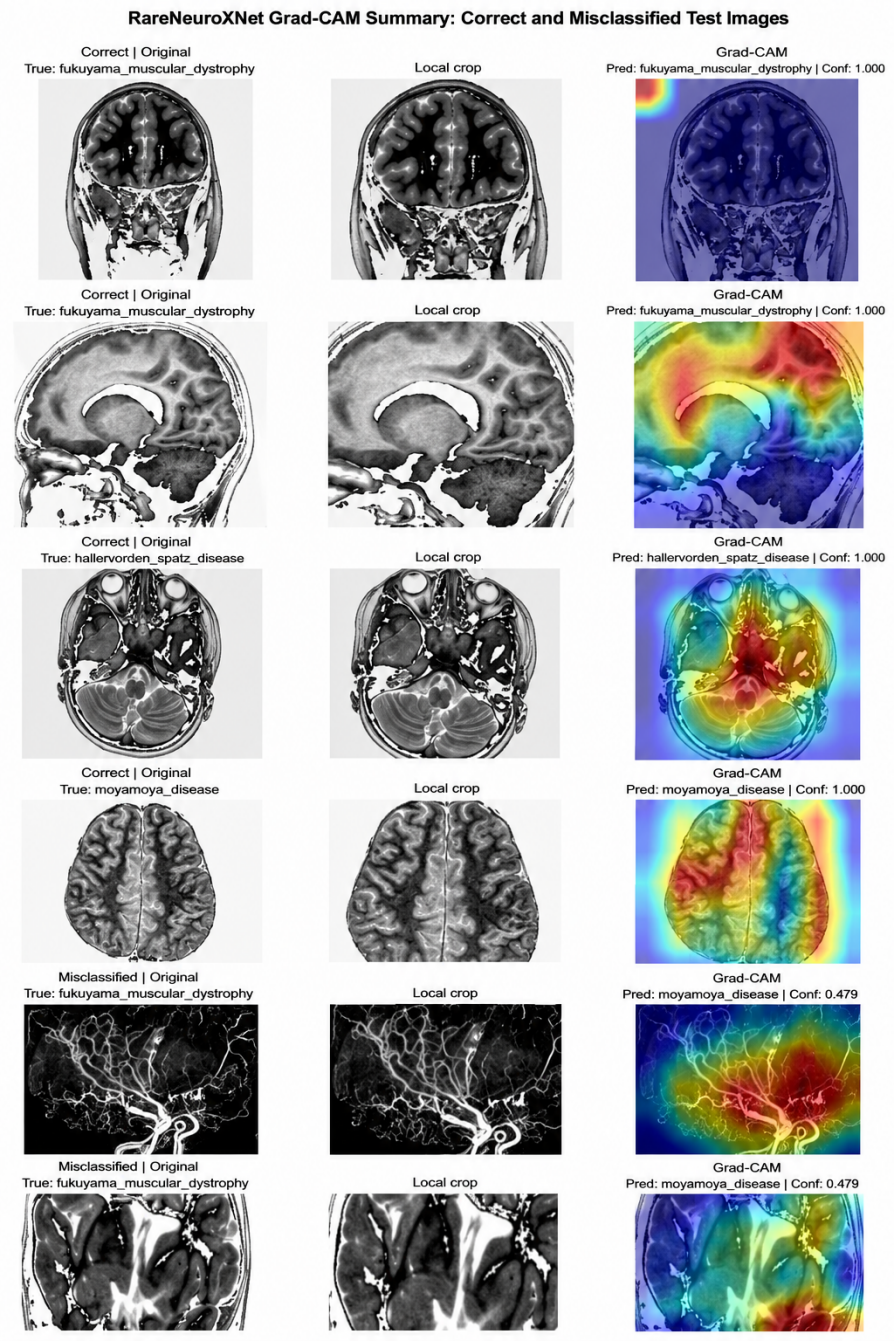
Grad-CAM visual explanations of RareNeuroXNet predictions.

Grad-CAM was used to provide qualitative insight into the decision behavior of RareNeuroXNet. Heatmaps were generated for representative correctly classified and misclassified test images by backpropagating class-specific gradients to the final convolutional feature maps. The purpose was to examine whether the model relied on localized intracranial regions rather than non-informative areas such as image borders, background, skull regions, or preprocessing artifacts. As shown in Figure 12, correctly classified examples generally highlighted structurally informative brain regions, supporting the importance of local feature learning observed in the ablation study. In contrast, the misclassified example showed a more diffuse activation pattern and lower prediction confidence, suggesting greater uncertainty in the model decision. Although these visualizations provide useful interpretability evidence, Grad-CAM remains qualitative and does not confirm clinical correctness. Future work should include expert radiological review and complementary explainability methods such as saliency maps or occlusion sensitivity.

Figure 12 shows representative visualizations of Grad-CAM for correct and incorrect predictions of the proposed model RareNeuroXNet on the test set. Each row presents the original MRI image, the local image cropped by the local branch, and the Grad-CAM heatmap applied to the cropped image. Examples are correctly classified cases of Fukuyama muscular dystrophy, Hallervorden–Spatz disease, and Moyamoya disease, as well as a misclassified case in which an image of Fukuyama muscular dystrophy was classified as Moyamoya disease. The warmer colors represent areas that were more strongly affiliated with the predicted class, and the cooler colors represent lower affiliation.

The visualizations from the Grad-CAM method give qualitative information about the decision-making process employed by RareNeuroXNet. For the well-classed examples, the heatmaps typically focus on the local intracranial regions and structurally informative brain areas and not just on the image borders and/or background regions. This is consistent with the ablation results, which demonstrated the most important part of the model for the local branch. The highlighted areas differ in anatomical view and disease classes, indicating that the model relies on small-scale structural and textural detail for its prediction. For instance, the examples correctly classified as Hallervorden–Spatz and Moyamoya demonstrate focus on the center of the image or the area relevant to the disease, whereas the local crop assists in highlighting the brain area of interest instead of the background of the image. The misclassified example exhibits more irregular behavior: although the model is still able to mark out salient anatomical regions, the focus is less concentrated and partially in anatomical regions that more or less clearly correspond to the actual class-specific pattern. It also has a lower CV than the correctly classified examples, indicating more uncertainty. Overall, the number aligns with the potential interpretability of the proposed local-feature learning strategy; however, Grad-CAM is still a qualitative tool and should be used with caution. The areas of interest should be reviewed by an expert radiologist to confirm the presence of clinically significant disease patterns.

#### 4.5.3. Baseline Comparison

A variant of the standard architecture was also proposed, and several baseline models were trained with the same dataset partition, input resolution, augmentation strategy, optimizer settings, and evaluation protocol to assess whether the proposed multi-branch design offers a meaningful advantage over the standard architectures. EfficientNetB0, DenseNet121, ResNet50, local-crop-only DenseNet121, and ViT-small transformer baselines were used. This comparison allows RareNeuroXNet to be compared to conventional CNN backbones, as well as to architecture relying on attention, while maintaining the same experimental conditions. The same metrics—accuracy, macro F1 score, macro AUROC, macro AUPR, ECE, and NLL—were used to evaluate all models.

The internal baseline comparison was performed on the curated rare neurologic disease MRI dataset using the same evaluation protocol, and the results are presented in Table 9. RareNeuroXNet outperformed the other models compared in terms of accuracy, macro-F1, macro-AUC, macro-AUPR, ECE, and NLL with values of 0.992380, 0.992360, 0.999780, 0.999240, 0.005220, and 0.027560, respectively, which shows that it has strong discriminative performance and good reliability in making probabilistic predictions. DenseNet121 local-only was the closest competing model, achieving an accuracy of 0.989400, macro-F1 of 0.989380, macro-AUC of 0.999300, and macro-AUPR of 0.998380. The ablation results demonstrate that the local branch has the most significant impact on the performance of DenseNet121, which fits with the small gap of RareNeuroXNet and DenseNet121 local-only in the local-only setting. The DenseNet121 model achieved a high accuracy of 0.976667, a macro-F1 score of 0.976638, a macro-AUC of 0.989847, and a macro-AUPR of 0.989402 as well, demonstrating the effectiveness of DenseNet-based representations for this task. EfficientNetB0 and ViT-Small Transformer, however, achieved significantly lower performance scores with accuracies of 0.346667 and 0.250000, respectively, and macro-F1 of 0.345708 and 0.166961, respectively. These less favorable results could be caused by a lack of compatibility of these architectures with the current dataset features or with the training setup or with the distribution of the features of the images in the dataset. In total, Table 6 indicates that DenseNet121 local-only is not far behind RareNeuroXNet of the full multi-branch architecture, suggesting this considerable local contribution to predictive performance. Hence, the roles played by the global or FFT branches need to be taken with a pinch of salt, and the small gap between RareNeuroXNet and DenseNet121 local-only requires fold-wise paired statistical testing before being declared as a clear statistical superiority.

#### 4.5.4. Paired Cross-Validation Comparison with the DenseNet121 Local-Only Baseline

Table 10 shows the performance of RareNeuroXNet, that compared with DenseNet121, the local-only baseline model, with the same five-fold cross-validation methodology. The classification and discrimination results for both models were very good, with the mean values being slightly higher for RareNeuroXNet. RareNeuroXNet obtained an accuracy, macro-F1, weighted-F1, precision macro, and recall macro of 0.9924 ± 0.0061, while DenseNet121 local-only obtained an accuracy, macro-F1, weighted-F1, precision macro and recall macro of 0.9894 ± 0.0045, 0.9896 ± 0.0044, and 0.9894 ± 0.0045, respectively. The discrimination metrics followed the same trend, with RareNeuroXNet achieving a macro-AUC of 0.9998 ± 0.0002 and a macro-AUPR of 0.9992 ± 0.0007, compared with 0.9993 ± 0.0008 and 0.9984 ± 0.0017 for DenseNet121 local-only, respectively. The results indicate that using the proposed multi-branch architecture along with local, global, and frequency-aware representations leads to a slight boost in classification accuracy and class separability. The calibration metrics also favored RareNeuroXNet, which achieved a lower ECE of 0.0052 ± 0.0029 and a lower NLL of 0.0276 ± 0.0159, compared with 0.0096 ± 0.0042 and 0.0460 ± 0.0146 for DenseNet121 local-only. This means that RareNeuroXNet had more accurate probability estimates and more accurate confidence calibration on all folds. Overall, the comparison revealed that RareNeuroXNet performed best on the mean of classification, discrimination, and calibration values, but DenseNet121 local-only was still a competitive baseline. Thus, the improvement of the complete RareNeuroXNet design is to be taken in the light of the strength of a good local feature extractor, not as a large performance increase, and the slight differences observed should be interpreted in the context of fold-wise paired statistical tests to assess whether these differences are statistically significant.

The paired fold-wise statistical comparison of RareNeuroXNet and a local-only baseline (DenseNet121) over the same five folds of cross-validation is reported in Table 11. The mean difference was defined as RareNeuroXNet – DenseNet121 local-only; therefore, positive values indicate higher classification performance for RareNeuroXNet, whereas negative values for ECE and NLL indicate lower calibration error and uncertainty for RareNeuroXNet. RareNeuroXNet achieved slightly higher mean values for the main classification metrics, improving accuracy from 0.9894 to 0.9924, macro-F1 from 0.9894 to 0.9924, weighted-F1 from 0.9894 to 0.9924, precision macro from 0.9896 to 0.9924, and recall macro from 0.9894 to 0.9924. The discrimination metrics also showed the same trend, with a rise from macro-AUC = 0.9993 to 0.9998, and macro-AUPR = 0.9984 to 0.9992. The improvements were modest, however, with the classification metric improvements being around 0.0028–0.0030, the macro-AUC improvement being around 0.0005, and the macro-AUPR improvement being around 0.0009. All *p*-values for accuracy, macro-F1, macro-AUC, macro-AUPR, weighted-F1, precision macro, and recall macro were greater than 0.05 for the paired *t*-test and Wilcoxon signed-rank test, and the results did not turn out to be statistically significant across the five folds. RareNeuroXNet, on the other hand, indicated more improvements in terms of calibration metrics. The ECE decreased from 0.0096 to 0.0052, with a mean difference of −0.0044, and NLL decreased from 0.0460 to 0.0276, with a mean difference of −0.0185. These negative differences are good for RareNeuroXNet, as they reflect a lower probability of calibration error and lower prediction uncertainty. Both the ECE and the NLL exhibited statistically significant differences with the local-only baseline, with *p* = 0.0416 and *p* = 0.0369, respectively, indicating better calibration. In both cases, however, Wilcoxon *p*-values of 0.1250 were not statistically significant. Table 8 shows that RareNeuroXNet, overall, had slightly superior mean classification, discrimination, and calibration performance compared to DenseNet121 local only, and the mean improvement in classification was small but non-statistically significant. The greatest benefit seen from RareNeuroXNet was during calibration, in support of the usefulness of the proposed multi-branch architecture for probabilistic reliability and in line with DenseNet121 local-only being a good baseline. These results should be interpreted with caution, as there are few folds, and they must be confirmed with larger datasets and external validation cohorts.

### 4.6. Discussion

The experimental results indicate that RareNeuroXNet achieved strong performance within a controlled image-level benchmark for rare neurological disease MRI classification. The classification results of the model were high and stable throughout the fivefold cross-validation experiments, with the accuracy, macro precision, macro recall, macro F1 score, and weighted F1 score ranging from 0.98 to 1.00. This stability was further confirmed by the mean cross-validation results, which showed that the accuracy of RareNeuroXNet was 0.9924 ± 0.0061, the macro F1-score was 0.9924 ± 0.0061, and the weighted F1-score was 0.9924 ± 0.0061. Also, there was high class separability for the ROC and PR analyses with macro AUROC and macro AUPR values of 0.9998 ± 0.0002 and 0.9992 ± 0.0007, respectively. These results indicate that the proposed multi-branch architecture is able to obtain highly discriminative image-level representation learning from the curated rare neurological MRI dataset.

But the near-perfect in-house performance must be interpreted cautiously. The main data set is curated, balanced, and evaluated at the image level. The input MRI data can be significantly different in terms of scanner make, acquisition protocol, field strength, image quality, slice selection, disease stage, and patient population. Furthermore, the public data set was not complete and did not contain full patient identifiers, and strict patient-level separation was not fully confirmed. Hence, it is possible to have an inflated performance estimate if correlated images, duplicate images, or almost identical images for the same subject are spread over multiple folds. Therefore, the reported results are to be viewed as a measure and index of good internal image-level benchmark performance and not as definitive evidence of patient-level or clinical generalization.

One good thing about this evaluation is that it consisted not just of the usual classification metrics, but also of probability-based reliability analysis. RareNeuroXNet achieved low calibration error, with an ECE of 0.0052 ± 0.0029 and an NLL of 0.0276 ± 0.0159. The values in the table indicate that the confidence estimates given by the model were well in line with the observed class labels in the evaluated benchmark. This is crucial since the goal of a medical image classification model is not only to correctly predict the class but to produce meaningful confidence estimation as well. However, calibration is susceptible to domain shift, and confidence behavior might shift if the model is deployed to images that were collected from a different institution, scanner, or protocol. So, recalibration and validation on the external patient level and multi-center datasets are still required prior to clinical translation.

The ablation analysis allowed us to gain insights into the relative contribution of the architectural components. The best accuracy, precision, recall, and F1 were obtained with the full RareNeuroXNet model, with accuracy, precision, recall, and F1 scores of 0.99 and ROC-AUC and PR-AUC scores of 1.00. The accuracy and F1-score dropped to 0.97 and 0.96, respectively, after removing the FFT branch and CBAM branch, respectively. The accuracy, precision, recall, and F1-score for the weighted fusion variant were competitive, at 0.98. The results indicate that FFT and CBAM can be helpful refinement and incremental improvements. Removal of the local branch, however, resulted in a significant drop in accuracy of 0.35, an F1 score of 0.31, an ROC AUC of 0.73, and a PR AUC of 0.43. The no-local variant also had weaker calibration, with calibration ECE rising to 0.06 and NLL rising to 1.43. The results validate that the local branch is the most powerful part of RareNeuroXNet for this tested dataset, whereas the global and FFT branches contribute comparatively less.

This is corroborated by the internal baseline comparison. Overall, RareNeuroXNet achieved the highest accuracy (0.992380), macro-F1 (0.992360), macro-AUC (0.999780), and macro-AUPR (0.999240) among all the models. DenseNet121 local-only was the closest competing model, achieving an accuracy of 0.989400, macro-F1 of 0.989380, macro-AUC of 0.999300, and macro-AUPR of 0.998380. It again validates that DenseNet-based local feature extraction is highly informative in the selected rare classes of neurological diseases and in agreement with the ablation result, which showed that the local branch is the major contributor. DenseNet121 had excellent results, while ResNet50 had moderate-to-good results. However, some widely used architectures were not as effective as EfficientNetB0 and the ViT-Small Transformer in the given context of images and their training, highlighting the fact that not every architecture is appropriate.

The paired statistical comparison of the local-only models RareNeuroXNet and DenseNet121 indicated slightly higher mean values for accuracy, macro-F1, macro-AUC, macro-AUPR, weighted-F1, precision macro, and recall macro for RareNeuroXNet. For instance, DenseNet121 local-only achieved an accuracy of 0.9894, while RareNeuroXNet obtained an accuracy of 0.9924, and the difference between the two means and standard mean difference was 0.0030. When measured by the main classification and discrimination measures, however, the paired *t*-test and Wilcoxon signed-rank test did not reveal any statistically significant differences. This suggests that the classification improvement is rather small relative to the strong local-only baseline model and should not be exaggerated. As a comparison, the ECE of RareNeuroXNet reduced from 0.0096 to 0.0052, and the NLL decreased from 0.0460 to 0.0276. The paired *t*-test showed significant differences for ECE but not for NLL, while the Wilcoxon test did not confirm significance for either. The most consistent benefit of RareNeuroXNet seems to be increased probabilistic reliability, rather than an increased rate of correct classification.

To serve as a complementary neurological MRI benchmark, the MCND dataset was used. MCND is not an external validation set applied on the same set of rare neurological disease classes as the same task, as it comprises several different stages of Alzheimer’s disease, brain tumor classes, multiple sclerosis, and normal cases. It should thus be understood as an extra mode of evaluation and not as external validation. The MCND results revealed that the performance of the model was different for different classes of disease, with better performance for certain classes, and errors were made between similar or related classes in terms of visual similarity or clinical symptoms. This confirms that cross-dataset benchmarking is useful and highlights the need for same-task, independent external validation with other rare neurological disease cohorts.

Grad-CAM visual explanation was added to obtain qualitative understanding of the image regions that are important for model predictions. The obtained heatmaps were mostly localized in the brain areas and were structurally informative, indicating the significance of local feature learning found in the ablation analysis. But Grad-CAM is still a qualitative explanation technique that cannot judge clinical correctness by itself. Therefore, the highlighted areas should be assessed by radiology specialists to identify if they are associated with clinically relevant disease patterns. Further research should also include other complementary explainability techniques like saliency maps, occlusion sensitivity, attention visualization, and expert-guided region validation.

However, there are some restrictions in input representation and dataset design. RareNeuroXNet currently focuses on 2D MRI images instead of 3D MRI volumes. This is a design style that is suitable for the public data set that is available, but the clinical neurological diagnosis may be based on volumetric context, the distribution of lesions across slices, and multimodal MRI imaging sequences like T1, T2, and FLAIR. The fixed center crop in the local branch should also be interpreted with care, since spatial regularities according to the dataset could appear if images are, e.g., aligned or preprocessed in the same way. In the future, the authors would like to compare center cropping to brain-mask-guided crops, random local crops, artifact-controlled preprocessing, and 3D multimodal architectures.

In summary, RareNeuroXNet is a potentially promising controlled benchmark-based system for brain MRI image-level classification of rare neurological diseases. Advantages of using it are the incorporation of global, local, and frequency domain representations; calibration-aware evaluation; visual explanations using Grad-CAM; and ablation evidence of the importance of local feature modeling. However, patient-level splitting, duplicate and near-duplicate image filtering, same-task external multi-center validation, reporting confidence intervals, repeated fold-wise ablation testing, and expert-validated explainability would be needed for stronger conclusions. Additionally, future studies should also test RareNeuroXNet through a thorough evaluation protocol on a patient-level scale as compared to larger transformer-based and/or foundation-model approaches.

## 5. Conclusions

To alleviate this problem, in this study, a frequency-aware multi-branch attention framework, named RareNeuroXNet, was proposed to provide image-level classification for rare neurological diseases in brain magnetic resonance imaging (MRI). The model combines FFT magnitude-based frequency information, global anatomical representation, and local fine-grained feature extraction in a single fusion architecture. The results of the experiments showed good image-level internal benchmark performance on the balanced curated dataset with a stable performance on the cross-validation with five folds. RareNeuroXNet achieved a mean accuracy of 0.9924 ± 0.0061, a macro F1-score of 0.9924 ± 0.0061, a weighted F1-score of 0.9924 ± 0.0061, a macro AUROC of 0.9998 ± 0.0002, and a macro AUPR of 0.9992 ± 0.0007. Moreover, the model exhibited desirable calibration performance measured by an ECE of 0.0052 ± 0.0029 and an NLL of 0.0276 ± 0.0159 within the benchmarked scale, within the curated image-level benchmark.

The ablation analysis revealed that the local branch was the most dominating part of the proposed system. The local branch deletion resulted in a significant drop in accuracy (0.35), F1-score (0.31), ROC-AUC (0.73), and PR-AUC (0.43) scores. This suggests within this image-level benchmark that fine-grained local MRI features are highly informative for the classification of rare neurological disease classes, which are visually similar. Removing the FFT branch and CBAM attention module, however, resulted in smaller decreases in performance, indicating that they contribute complementary refinement to the performance, rather than being the primary ones. The full RareNeuroXNet model configuration also performed well; even though the weighted-fusion variant performed competitive results, the complete model exhibited the best overall balance of classification accuracy, discrimination, and calibration.

The internal baseline comparison also revealed that RareNeuroXNet had the best overall performance of all the models evaluated. However, DenseNet121 local-only was also a competitive baseline with an accuracy of 0.9894 versus 0.9924 for RareNeuroXNet. The paired statistical analysis revealed that the mean accuracy, mean macro-F1, mean macro-AUC, mean macro-AUPR, mean weighted-F1, mean macro-precision, and mean macro-recall were slightly higher for RareNeuroXNet than for DenseNet121 local-only, but these classification and discrimination gains were not statistically significant across the five folds. The clearest advantage of RareNeuroXNet was observed in calibration, where it reduced ECE from 0.0096 to 0.0052 and NLL from 0.0460 to 0.0276. For this reason, using the full multi-branch architecture should be viewed as an incremental improvement over a solid local DenseNet architecture, including probabilistic reliability.

To test the model’s performance on different classification tasks, an additional cross-dataset neurological MRI benchmark was created in the MCND experiment. MCND should not be viewed as the same task for external validation, as it includes Alzheimer’s disease stages and several different types of brain tumor, multiple sclerosis, and normal cases—not the same as in the primary experiment. Thus, MCND measures must not be used as evidence of external generalization to the target rare disease task but as complementary evidence of model behavior across neurological MRI classification settings.

It would be useful to acknowledge some of the limitations in interpreting the findings. The main data set is curated, image-level, and balanced and free of full patient identifiers, MRI acquisition metadata, scanner information, slice-per-patient counts, and independent same-task external validation cohorts. Thus, there was no evidence of strict separation by subject, and the excellent internal results may be misleading in the real world, as there is the possibility of correlated slices, duplicate or near-duplicate images, or overlap of subjects across partitions. Furthermore, the current framework relies on 2D MRI images as opposed to full 3D multimodal MRI volumes, which are not sufficiently able to capture volumetric patterns of disease, distribution of lesions across slices, and sequence-specific information from modalities including T1, T2, and FLAIR.

The next steps for this work involve patient-level splitting, duplicate and near-duplicate image screening, external multi-center validation with the same rare neurological disease classes and providing confidence intervals with the results. The framework should also be expanded to 3D multimodal MRI analysis and evaluated using the methods validated by experts as explainable. Lastly, a comparative study with CNN backbone, transformers, self-supervised pretrained networks, and medical foundation models would be required to assess whether RareNeuroXNet offers sustained benefits in larger, independent, and more clinically representative datasets. Therefore, the present findings should be regarded as image-level benchmark evidence rather than evidence of patient-level or clinical diagnostic generalization.

## Figures and Tables

**Table 1:** Summary of state of the arts.

Ref	Technique	Dataset	Accuracy	Main Limitation
Ismael et al., 2020 [3]	Residual-network transfer learning	Benchmark brain tumor MRI dataset	99.0%	Focused on common tumors
Chatterjee et al., 2022 [8]	Spatiotemporal ResNet variants	Brain tumor MRI dataset	96.98%	Tumor-only setting
Siddiqi et al., 2022 [9]	Enhancement + wavelet + ML	Brain MRI disease dataset	96.6%	Less flexible than end-to-end DL
Ahmed et al., 2024 [11]	ViT-GRU hybrid	BrTMHD-2023 primary dataset	98.97%	Evaluated on tumor data
Jayaraman et al., 2025 [14]	CNN–ViT cross-attention fusion	Not reliably verified from accessible source used in this session	96.41%	Common-disease benchmark
Ahmed et al., 2025 [16]	Swin Transformer + ResNet	Not reliably verified from accessible source used in this session	99.79%	Curated tumor-only data
Moshe et al., 2026 [32]	DDPM + mutual information + ensemble	Two datasets (“Dataset I” and “Dataset II”) in accessible summary	>98% on Dataset I, and 93.18% on Dataset II	Tumor-focused
Khan et al., 2025 [33]	Hybrid deep learning	Brain tumor MRI benchmark	99.11%	Limited rare-disease applicability

**Table 2:** Dataset distribution.

Disease Class	Train	Validation	Test	Total
Fukuyama muscular dystrophy	280	60	60	400
Hallervorden–Spatz disease	280	60	60	400
Moyamoya disease	280	60	60	400
Pachygyria with cerebellar hypoplasia	280	60	60	400
Walker–Warburg syndrome	280	60	60	400
Total	1400	300	300	2000

**Table 3:** Cross Validation classification reports.

Metric	Fold 1	Fold 2	Fold 3	Fold 4	Fold 5
accuracy	0.99	1.00	0.98	0.99	1.00
precision_macro	0.99	1.00	0.98	0.99	1.00
recall_macro	0.99	1.00	0.98	0.99	1.00
f1_macro	0.99	1.00	0.98	0.99	1.00
f1_weighted	0.99	1.00	0.98	0.99	1.00
ece	0.01	0.00	0.00	0.01	0.00
nll	0.03	0.01	0.05	0.03	0.02

**Table 4:** Mean and standard deviation of the cross-validation performance metrics.

Metric	Mean	Std
accuracy	0.9924	0.0061
f1_macro	0.9924	0.0061
f1_weighted	0.9924	0.0061
roc_auc_macro_ovr	0.9998	0.0002
pr_auc_macro	0.9992	0.0007
ece	0.0052	0.0029
nll	0.0276	0.0159

**Table 5:** Ablation Results.

Model	Acc.	Prec.	Rec.	F1	ECE	NLL	ROC-AUC	PR-AUC
noFFT	0.97	0.97	0.96	0.97	0.03	0.04	1.00	1.00
noCBAM	0.96	0.96	0.95	0.96	0.04	0.04	1.00	1.00
weightedFusion	0.98	0.98	0.98	0.98	0.04	0.04	1.00	1.00
noLocal	0.35	0.38	0.35	0.31	0.06	1.43	0.73	0.43
full	0.99	0.99	0.99	0.99	0.01	0.03	1.00	1.00

**Table 6:** Mean and standard deviation of five-fold cross-validation results for RareNeuroXNet, ablation variants, and the DenseNet121 local-only baseline.

Metric	Full	noFFT	noCBAM	weightedFusion	noLocal	DenseNet121
Accuracy mean	0.9924	0.9206	0.8853	0.9176	0.3412	0.8882
Accuracy std	0.0061	0.0189	0.0230	0.0106	0.0398	0.0083
F1 macro mean	0.9924	0.9200	0.8851	0.9171	0.2779	0.8877
F1 macro std	0.0061	0.0192	0.0229	0.0111	0.0513	0.0086
ROC-AUC macro mean	0.9998	0.9924	0.9844	0.9926	0.6665	0.9858
ROC-AUC macro std	0.0002	0.0022	0.0038	0.0022	0.0314	0.0030
PR-AUC macro mean	0.9992	0.9752	0.9525	0.9760	0.3610	0.9556
PR-AUC macro std	0.0007	0.0060	0.0121	0.0057	0.0346	0.0074
ECE mean	0.0052	0.0345	0.0661	0.0285	0.1077	0.0563
ECE std	0.0029	0.0083	0.0188	0.0046	0.0361	0.0160
NLL mean	0.0276	0.2297	0.3471	0.2287	1.5574	0.3301
NLL std	0.0159	0.0356	0.0372	0.0296	0.0168	0.0345

**Table 7:** Fold-wise across five-fold cross-validation.

Model	Fold	Accuracy	F1-Macro	ROC-AUC	PR-AUC	ECE	NLL
full	1	0.9918	0.9922	0.9998	0.9992	0.0100	0.0300
full	2	1.0000	1.0000	0.9998	0.9992	0.0000	0.0100
full	3	0.9853	0.9851	0.9998	0.9992	0.0000	0.0500
full	4	0.9976	0.9966	0.9998	0.9992	0.0100	0.0300
full	5	1.0000	1.0000	0.9998	0.9992	0.0000	0.0200
noFFT	1	0.9088	0.9074	0.9898	0.9693	0.0265	0.2750
noFFT	2	0.9059	0.9049	0.9906	0.9699	0.0461	0.2501
noFFT	3	0.9382	0.9377	0.9942	0.9809	0.0367	0.2036
noFFT	4	0.9059	0.9060	0.9926	0.9740	0.0264	0.2335
noFFT	5	0.9441	0.9440	0.9948	0.9820	0.0366	0.1862
noCBAM	1	0.8824	0.8819	0.9830	0.9494	0.0660	0.3626
noCBAM	2	0.8882	0.8881	0.9878	0.9644	0.0424	0.3223
noCBAM	3	0.9176	0.9174	0.9891	0.9656	0.0811	0.3009
noCBAM	4	0.8529	0.8529	0.9821	0.9383	0.0536	0.3523
noCBAM	5	0.8853	0.8852	0.9801	0.9449	0.0876	0.3972
weightedFusion	1	0.9059	0.9049	0.9890	0.9667	0.0359	0.2774
weightedFusion	2	0.9324	0.9321	0.9946	0.9817	0.0233	0.2030
weightedFusion	3	0.9088	0.9076	0.9926	0.9773	0.0262	0.2319
weightedFusion	4	0.9206	0.9199	0.9942	0.9788	0.0288	0.2078
weightedFusion	5	0.9206	0.9209	0.9927	0.9756	0.0283	0.2236
noLocal	1	0.3794	0.3440	0.6806	0.3856	0.1343	1.5331
noLocal	2	0.3265	0.2491	0.6381	0.3287	0.0930	1.5663
noLocal	3	0.3676	0.3041	0.6717	0.3556	0.1437	1.5635
noLocal	4	0.3529	0.2823	0.7088	0.4063	0.1143	1.5484
noLocal	5	0.2794	0.2100	0.6332	0.3285	0.0534	1.5758
DenseNet121 local-only	1	0.8912	0.8897	0.9806	0.9427	0.0812	0.3848
DenseNet121 local-only	2	0.8941	0.8941	0.9876	0.9601	0.0483	0.2976
DenseNet121 local-only	3	0.8971	0.8970	0.9873	0.9610	0.0623	0.3073
DenseNet121 local-only	4	0.8794	0.8788	0.9871	0.9569	0.0405	0.3203
DenseNet121 local-only	5	0.8794	0.8786	0.9862	0.9572	0.0494	0.3403

**Table 8:** Paired fold-wise statistical comparison between the full RareNeuroXNet model and ablation variants across five-fold cross-validation.

Comparison	Metric	Full Mean	Variant Mean	Mean Difference	Std Difference	Paired *t*-Test *p*-Value	Wilcoxon *p*-Value
full vs. noFFT	Accuracy	0.9924	0.9206	0.0718	0.0217	0.0018	0.0625
full vs. noFFT	F1-macro	0.9924	0.9200	0.0724	0.0220	0.0018	0.0625
full vs. noFFT	ECE	0.0052	0.0345	−0.0293	0.0134	0.0070	0.0625
full vs. noFFT	NLL	0.0276	0.2297	−0.2021	0.0416	0.0004	0.0625
full vs. noCBAM	Accuracy	0.9924	0.8853	0.1071	0.0273	0.0009	0.0625
full vs. noCBAM	F1-macro	0.9924	0.8851	0.1073	0.0272	0.0009	0.0625
full vs. noCBAM	ECE	0.0052	0.0661	−0.0609	0.0211	0.0027	0.0625
full vs. noCBAM	NLL	0.0276	0.3471	−0.3195	0.0455	<0.0001	0.0625
full vs. weightedFusion	Accuracy	0.9924	0.9176	0.0748	0.0071	<0.0001	0.0625
full vs. weightedFusion	F1-macro	0.9924	0.9171	0.0753	0.0071	<0.0001	0.0625
full vs. weightedFusion	ECE	0.0052	0.0285	−0.0233	0.0036	0.0001	0.0625
full vs. weightedFusion	NLL	0.0276	0.2287	−0.2011	0.0280	<0.0001	0.0625
full vs. noLocal	Accuracy	0.9924	0.3412	0.6512	0.0465	<0.0001	0.0625
full vs. noLocal	F1-macro	0.9924	0.2779	0.7145	0.0576	<0.0001	0.0625
full vs. noLocal	ECE	0.0052	0.1077	−0.1025	0.0342	0.0025	0.0625
full vs. noLocal	NLL	0.0276	1.5574	−1.5298	0.0249	<0.0001	0.0625
full vs. DenseNet121	Accuracy	0.9924	0.8882	0.1042	0.0141	<0.0001	0.0625
full vs. DenseNet121	F1-macro	0.9924	0.8877	0.1047	0.0143	<0.0001	0.0625
full vs. DenseNet121	ECE	0.0052	0.0563	−0.0511	0.0155	0.0016	0.0625
full vs. DenseNet121	NLL	0.0276	0.3301	−0.3025	0.0370	<0.0001	0.0625

**Table 9:** Performance comparison of RareNeuroXNet with internal baseline models using the curated rare neurological disease MRI dataset.

Model	Accuracy	Macro-F1	Macro-AUC	Macro-AUPR	ECE	NLL	Weighted-F1	Precision_Macro	Recall_Macro	Temperature
EfficientNetB0	0.346667	0.259390	0.662819	0.342490	0.031242	1.491701	0.259390	0.230890	0.346667	1.116582
DenseNet121	0.976667	0.976638	0.989847	0.989402	0.002706	0.019403	0.976638	0.976776	0.976667	0.490000
ResNet50	0.840000	0.837005	0.973292	0.938978	0.030463	0.417369	0.837005	0.860063	0.840000	0.945696
DenseNet121 local-only	0.989400	0.989380	0.999300	0.998380	0.009640	0.046020	0.989380	0.989580	0.989400	0.812240
ViT-Small Transformer	0.250000	0.198006	0.594917	0.264568	0.004411	1.552806	0.198006	0.198993	0.250000	1.230506
RareNeuroXNet	0.992380	0.992360	0.999780	0.999240	0.005220	0.027560	0.992360	0.992400	0.992420	0.610940

**Table 10:** Five-fold cross-validation comparison between RareNeuroXNet and the DenseNet121 local-only baseline.

Model	Accuracy	Macro-F1	Macro-AUC	Macro-AUPR	ECE	NLL	Weighted-F1	Precision Macro	Recall Macro
RareNeuroXNet	0.9924 ± 0.0061	0.9924 ± 0.0061	0.9998 ± 0.0002	0.9992 ± 0.0007	0.0052 ± 0.0029	0.0276 ± 0.0159	0.9924 ± 0.0061	0.9924 ± 0.0061	0.9924 ± 0.0061
DenseNet121 local-only	0.9894 ± 0.0045	0.9894 ± 0.0045	0.9993 ± 0.0008	0.9984 ± 0.0017	0.0096 ± 0.0042	0.0460 ± 0.0146	0.9894 ± 0.0045	0.9896 ± 0.0044	0.9894 ± 0.0045

**Table 11:** Paired statistical comparison between RareNeuroXNet and DenseNet121 local-only.

Metric	RareNeuroXNet Mean	DenseNet121 Local-Only Mean	Mean Difference	Std Difference	Paired *t*-Statistic	Paired *t*-Test *p*-Value	Wilcoxon Statistic	Wilcoxon *p*-Value
Accuracy	0.9924	0.9894	0.0030	0.0047	1.4286	0.2263	1.0000	0.2500
Macro-F1	0.9924	0.9894	0.0030	0.0047	1.4286	0.2263	1.0000	0.2500
Macro-AUC	0.9998	0.9993	0.0005	0.0007	1.4927	0.2098	1.5000	0.3750
Macro-AUPR	0.9992	0.9984	0.0009	0.0014	1.3831	0.2388	4.0000	0.4375
ECE	0.0052	0.0096	−0.0044	0.0033	−2.9583	0.0416	1.0000	0.1250
NLL	0.0276	0.0460	−0.0185	0.0134	−3.0805	0.0369	1.0000	0.1250
Weighted-F1	0.9924	0.9894	0.0030	0.0047	1.4286	0.2263	1.0000	0.2500
Precision Macro	0.9924	0.9896	0.0028	0.0046	1.3743	0.2413	2.0000	0.3750
Recall Macro	0.9924	0.9894	0.0030	0.0047	1.4286	0.2263	1.0000	0.2500

## Data Availability

https://www.kaggle.com/datasets/ahsanneural/rare-neurological-diseases-mri-curated-edition/data (accessed on 5 March 2026), https://www.kaggle.com/datasets/alifatahi/multi-class-neurological-disorder-mcnd-dataset (accessed on 15 March 2026).
